# Ocular complications in psoriatic patients: a systematic review and meta-analysis

**DOI:** 10.1186/s12348-025-00486-6

**Published:** 2025-03-17

**Authors:** Adriano Cypriano Faneli, Dillan Cunha Amaral, Isabelle Rodrigues Menezes, Guilherme Nunes Marques, Jaime Guedes, Rodrigo Brazuna, Ricardo Danilo Chagas Oliveira, Cristina Muccioli

**Affiliations:** 1 Bahiana School of Public Health and Medicine, Av. Dom João, 275, Salvador, BA 40290-000 Brazil; 2 Faculty of Medicine, Federal University of Rio de Janeiro, Rio de Janeiro, RJ Brazil; 3 State University of Rio Grande do Norte, Mossoró, RN Brazil; 4 Glaucoma Research Center, Wills Eye Hospital, Philadelphia, PA USA; 5 Federal University of the State of Rio de Janeiro, Rio de Janeiro, Brazil; 6 Federal University of Bahia, Salvador, BA Brazil; 7 Federal University of São Paulo, São Paulo, SP Brazil

**Keywords:** Meta-analysis, Systematic review, Eye disease, Psoriasis

## Abstract

**Purpose:**

To assess the prevalence of ocular findings in patients with psoriasis and compare the odds of developing these conditions between the psoriatic and control population through a systematic review and meta-analysis.

**Methods:**

A comprehensive literature search was conducted using PubMed, Embase, Cochrane, and Web of Science databases to identify studies reporting ocular findings in psoriasis patients. Inclusion criteria encompassed cross-sectional, case-control, cohort studies, case series, and case studies. Data extraction and quality assessment followed PRISMA guidelines. The Newcastle-Ottawa Scale evaluated the risk of bias. Heterogeneity was assessed using Cochran’s Q-test and I² statistics, with a random-effects model applied where significant heterogeneity was present.

**Results:**

30 studies comprising 131,687 patients (13,788 with psoriasis and 117,899 controls) were included. The relative likelihood of ocular findings in psoriasis patients showed to be increased in conjunctival hyperemia (OR = 7.38; 95% CI: 2.47–22.04), conjunctivitis (OR = 4.63; 95% CI: 1.42–15.08), dry eye (OR = 3.47; 95% CI: 2.06–5.83), and meibomian gland dysfunction (OR = 7.13; 95% CI: 2.14–23.72) compared to controls. In contrast, blepharitis, cataracts, episcleritis, glaucoma, pinguecula, pterygium, and uveitis did not differ significantly between the two groups.

**Conclusions:**

Psoriasis patients are at increased risk for certain ocular conditions, particularly conjunctival hyperemia, conjunctivitis, dry eye, and meibomian gland dysfunction. Further research is needed to understand the underlying mechanisms and to develop targeted management strategies.

**Supplementary Information:**

The online version contains supplementary material available at 10.1186/s12348-025-00486-6.

## Introduction

Psoriasis is a chronic, systemic inflammatory condition affecting the skin. It is driven by T-cell activity, which results in abnormal keratinocyte differentiation. This persistent disorder is primarily marked by skin manifestations influenced by genetic and environmental factors through the immune system. It impacts approximately 1–3% of the global population, with the highest incidence occurring in individuals aged 20–30 and 50–60 [1]. 

The pathogenesis of psoriasis is still not fully understood; however, significant attention has been given to T cell activation and increased activity of pro-inflammatory cytokines, especially tumor necrosis factor (TNF)-alfa. Besides skin lesions, clinical symptoms often include non-cutaneous manifestations such as depression, arthritis, uveitis, and cardiovascular diseases [2]. The worldwide high prevalence.

of psoriasis has led to the identification of psoriasis association with multiple comorbidities, such as cardiovascular disease, diabetes mellitus, and cancer [3]. Inflammatory eye changes are frequently observed in patients with immune-mediated conditions, particularly those with rheumatic diseases such as psoriatic arthritis (PsA). The average prevalence of ocular symptoms in psoriasis patients is around 10%. These ocular effects can impact various eye parts, including the cornea, lens, and eyelid [4]. 

Previous studies have reported the prevalence and relative likelihood of various ocular findings in patients with psoriasis. However, the overall prevalence and odds ratios of ocular conditions across all studies in psoriatic patients have not been summarized. Therefore, this systematic review and meta-analysis were conducted to assess the prevalence and relative likelihood of ocular findings in the psoriatic population and better elucidate the association between ophthalmological diseases and psoriasis.

## Methods

The protocol for this systematic literature review on ocular findings in patients with psoriasis was registered in the PROSPERO International Prospective Register of Systematic Review (CRD42024550012). We followed the Preferred Reporting Items for Systematic Review and Meta-Analysis (PRISMA) guidelines for data extraction [5]. The terms (“Palmoplantaris Pustulosis” OR Psoriasis OR “Pustular Psoriasis of Palms and Soles” OR “Pustulosis Palmaris et Plantaris” OR psoriatic) AND (eye OR ocular OR visual OR Ophthalmic OR optical OR ophthalmological) AND (symptom OR manifestation OR sign OR indicators OR issues OR presentation OR effects OR complications OR expressions OR anomalies OR symptoms OR manifestations OR signs OR presentations) were used for the search. Search terms were used to query PubMed, Embase, Cochrane, and Web of Science. The searches started in May 2024 and ended in June 2024. References from all the included studies, previous systematic reviews, and meta-analyses were manually searched for additional studies. Two authors (A.F. and D.A.) independently extracted data using predefined search criteria and quality assessment. The full articles of eligible publications were then scrutinized.

Only studies that met the following criteria were included in the meta-analysis.

Studies were included if they were cross-sectional, case-control, case series, case studies, or cohort studies. Commentaries or review articles were excluded. Any discrepancies between the two reviewers were discussed with another reviewer (I.M.) Two investigators (G.M. and I.M.) independently used a standardized form for data extraction, where any disagreements were discussed, and a third investigator (A.F.) was consulted if necessary. Data from all reported outcomes from the studies analyzed were collected. The prevalence of ocular diseases in psoriatic patients was predefined as the primary outcome of the analysis.

A Single-arm analysis was conducted to estimate the prevalence of ocular diseases in psoriatic patients. Additionally, a secondary analysis compared the prevalence of these diseases in psoriatic patients with uveitis to those without uveitis or with a history of uveitis, specifically for the uveitis analysis. There was no sufficient data for statistical analysis of all outcomes in the data collection. In this context the following ocular conditions were analyzed: Blepharitis, Cataracts, Conjunctivitis, Corneal opacity, Dry eye, Elevated intraocular pressure, Episcleritis, Glaucoma, Iritis, Keratitis, Lid margin abnormalities, Low visual acuity, Macular edema, Meibomian gland dysfunction, Pinguecula, Pseudophakia, Pterygium, Retinal detachment, Retinopathy, Trichiasis, Uveitis, Vitritis.

We also analyzed the relative likelihood, represented by the odds ratio, of psoriatic patients developing the following diseases compared to a non-psoriatic control population: Blepharitis, Cataracts, Conjunctival hyperemia, Conjunctivitis, Dry eye, Episcleritis, Glaucoma, Meibomian gland dysfunction, Pinguecula, Pterygium, Uveitis.

A quality assessment was performed by the two investigators who extracted the data. The Newcastle-Ottawa Scale was used to assess the risk of bias for cohort and case-control studies. In contrast, a modified Newcastle-Ottawa Scale was used to evaluate the risk for cross-sectional studies [6]. 

This systematic review and meta-analysis were performed using the Cochrane Collaboration and PRISMA statement guidelines [5]. Relative risk (RR) with 95% confidence intervals was used to compare treatment effects for categorical outcomes. Continuous outcomes were compared using standardized mean differences (SMD). Outcomes were measured as the mean ± standard deviation (SD). Statistical significance was set at 0.05. Cochran’s Q-test and I^2^ statistics were used to assess heterogeneity, and I^2^ > 50% indicated substantial heterogeneity. We used a random-effects model for outcomes with significant heterogeneity. Statistical analysis was performed using the R software (version 4.2.3, R Foundation for Statistical Computing, Vienna, Austria).

## Results

**Fig. 1:**
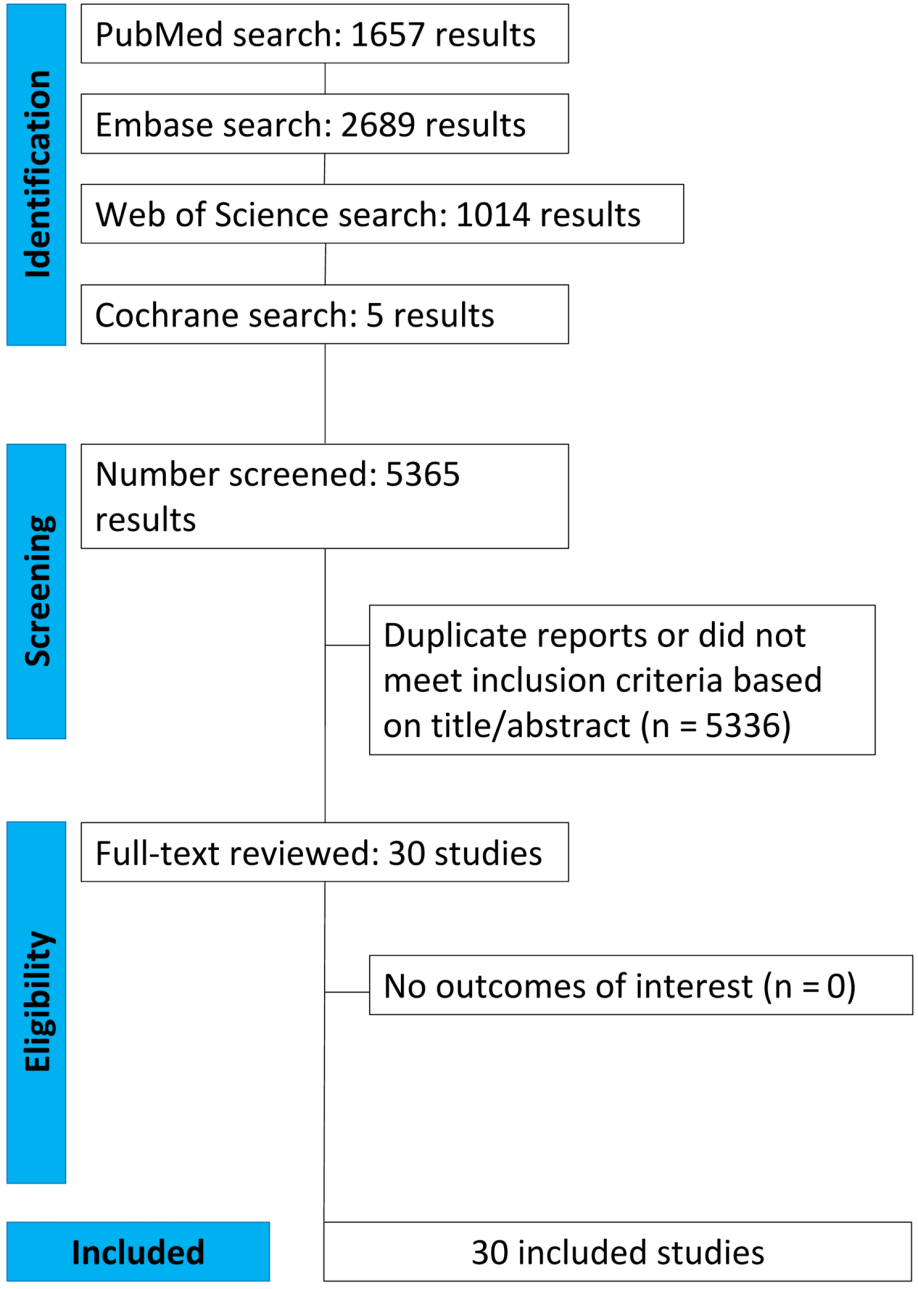
Flow diagram of study selection

As shown in Fig. 1, we found 5365 articles, with 1657 articles in PubMed, 2689 in Embase, 1014 in Web of Science, and 5 in Cochrane databases. Of these, 5335 were duplicates or did not meet inclusion criteria and were excluded from the analysis. Thirty studies remained and were thoroughly reviewed based on inclusion criteria. Ultimately, all 30 selected studies were included in this review, including five Case-Control studies [7–11], seventeen Cross-sectional studies [12–28], six non-randomized cohorts [29–34], and two case series [35, 36].

We analyzed 131,687 patients, 13,788 patients diagnosed with psoriasis, and 117,899 controls. The sample sizes of our studies ranged from 6 to 129,019. Follow-up period ranged from 1 month to 186 months. The characteristics of the studies are shown in Table 1.

**Table 1:** Baseline characteristics of the studies included

Study	Year	Study Design	Number of Patients	Psoriatic Patients	Control Patients	Age (Psoriatic Patients)	Age (Control Patients)	% Female (Psoriatic Patients)	% Female (Control Patients)	Follow up (months)	NOS Overall Star Rating
Abbouda	2016	Case-control	117	117	0	52.5 ± 16.0	NA	49.57%	NA	NA	5
Allen	2022	Cross-sectional	23	23	0	57.7 ± 14.8	NA	69.56%	NA	NA	5
Aragona	2018	Cross-sectional	94	66	28	50.7 ± 13.1	45.6 ± 16.3	50.00%	60.71%	NA	5
Aryanian	2022	Cross-sectional	80	40	40	45.38 ± 16.42	41.85 ± 14.96	60.00%	55.00%	NA	7
Campanati	2014	Cohort	64	32	32	52.2 ± 12.4	51.2 ± 12.3	37.50%	34.38%	3	8
Catsarou- Catsari	1984	Cross-sectional	101	101	0	NA	NA	35.64%	NA	NA	5
Dai	2021	Cohort	129.019	11.729	117.290	43.3	31.7–55.4	39.32%	39.32%	NA	6
da Cruz	2018	Cross-sectional	129	43	86	47.88 ± 14.61	47.47 ± 15.36	48.84%	53.49%	NA	6
de Lima	2012	Cross-sectional	80	40	40	53.9 ± 13.1	58.6 ± 12.5	60.00%	60.00%	NA	7
Demirci	2017	Cross-sectional	60	30	30	33.9 ± 5.1	33.8 ± 5	50.00%	53.33%	NA	5
Durrani	2005	Case-control	96	36	60	48.0 ± 17.4	NA	55.56%	33.33%	8.2 ± 4.7	6
Erbagci	2003	Case-control	61	31	30	39.2 ± 14.6	36.5 ± 14.2	51.61%	50.00%	NA	9
Gonzalez-Mazon	2019	Cross-sectional	406	406	0	46.3 ± 12.3	NA	49.75%	NA	120 ± 94.8	4
Goh	2021	Case-control	100	60	40	44 ± 17	41 ± 15	51.67%	37.50%	NA	6
Jain	1987	Cross-sectional	90	90	0	6–68	NA	23.33%	NA	NA	4
Kilic	2013	Cross-sectional	200	100	100	40.27 ± 12.24	40.49 ± 10.37	52.00%	52.00%	NA	7
Kemeriz	2022	Case-control	100	50	50	43,4 ± 14,1	41.2 ± 8.1	52.00%	50.00%	NA	7
Kharolia	2022	Cohort	68	68	0	43,69 ± 1,55	NA	20.59%	NA	2 months	6
Knox	1979	Case Series	10	10	0	32–68	NA	30.00%	NA	192	3
Lambert	1976	Cross-sectional	112	112	0	NA	NA	56.25%	NA	NA	4
Lee	2021	Cohort	20	20	0	50.1 ± 13.2	NA	45.00%	NA	46.8 ± 48	5
Oliveira	2024	Cross-sectional	130	130	0	50.7 ± 13.4	NA	46.92%	NA	NA	6
Sahin	2021	Cross-sectional	66	33	33	23–45	27–40	51.52%	51.52%	NA	6
Salek	2017	Case Series	6	6	0	2–12	NA	33.33%	NA	51–143	3
Sativada	2022	Cross-sectional	80	80	0	18–76	NA	36.25%	NA	NA	6
Singh	2022	Cross-sectional	126	126	0	42,39 ± 2,37	NA	38.10%	NA	NA	4
Subramanian	2021	Cross-sectional	105	105	0	50.3 (40–60)	NA	42.86%	NA	NA	4
Tanaka	2015	Cohort	13	13	0	38–58	NA	23.08%	NA	1-186	5
Tolba	2022	Cross-sectional	80	40	40	PS 41.4 ± 16.1 PMS 43.0 ± 9.8	35.8 ± 10.7	20.00%	55.00%	NA	6
Yang	2016	Cohort	51	51	0	NA	NA	27.45%	NA	1–57	5

## Single-Arm prevalence analysis

### Prevalence of anterior chamber flare

Anterior chamber flare prevalence was recorded and analyzed in patients with psoriasis, divided into subgroups with and without uveitis. High heterogeneity was observed between studies (*P* < 0.01, I² = 100%).

## Prevalence of blepharitis

Blepharitis prevalence was evaluated in psoriasis patients and categorized into subgroups based on the presence or absence of uveitis. In patients with uveitis, the proportion of blepharitis was relatively low (Proportion = 0.07; 95% CI: 0.03, 0.13; Supplementary Material Fig. 1). Conversely, the proportion was notably higher in patients without uveitis, with a combined effect model proportion of 0.20 (95% CI: 0.17, 0.22; Supplementary Material Fig. 1). High heterogeneity was present (*P* < 0.01, I² = 87%).

## Prevalence of cataracts

The prevalence of cataracts was assessed in psoriasis patients, grouped by the presence or absence of uveitis. In the uveitis subgroup, the proportion of cataracts was substantially higher (Proportion = 0.54; 95% CI: 0.46, 0.61; Supplementary Material Fig. 2) compared to those without uveitis (Proportion = 0.11; 95% CI: 0.09, 0.13; Supplementary Material Fig. 2). The studies displayed high heterogeneity (*P* < 0.01, I² = 95%).

### Prevalence of conjunctivitis

The prevalence of conjunctivitis was analyzed in psoriasis patients. The overall proportion of conjunctivitis was relatively low (Proportion = 0.04; 95% CI: 0.03, 0.06; Supplementary Material Fig. 3). The studies exhibited high heterogeneity (*P* < 0.01, I² = 93%).

## Prevalence of corneal opacity

The prevalence of corneal opacity was evaluated in psoriasis patients, stratified by the presence of uveitis. In the subgroup without uveitis, the proportion of corneal opacity was low (Proportion = 0.07; 95% CI: 0.01, 0.14; Supplementary Material Fig. 4). In patients with a diagnosis of uveitis, the proportion was similarly low (Proportion = 0.05; 95% CI: 0.03, 0.07; Supplementary Material Fig. 4). The studies exhibited moderate heterogeneity (*P* < 0.01, I² = 73%).

## Prevalence of dry eye

The prevalence of dry eye was assessed in psoriasis patients, divided into subgroups based on the presence or absence of uveitis. In the uveitis subgroup, the proportion of dry eye was moderate (Proportion = 0.18; 95% CI: 0.11, 0.26; Supplementary Material Fig. 5). For patients without a history of uveitis, the proportion was significantly higher, with a combined effect model proportion of 0.34 (95% CI: 0.31, 0.37; Supplementary Material Fig. 5). The analysis demonstrated high heterogeneity (*P* < 0.01, I² = 83%).

### Prevalence of elevated intraocular pressure

The prevalence of elevated intraocular pressure was evaluated in psoriasis patients, categorized by the presence of uveitis. In the uveitis subgroup, the proportion of elevated intraocular pressure was 0.23 (95% CI: 0.16, 0.32; Supplementary Material Fig. 6). For patients without uveitis, the proportion was slightly higher at 0.30 (95% CI: 0.17, 0.46; Supplementary Material Fig. 6). The studies exhibited no heterogeneity (*P* = 0.37, I² = 0%).

### Prevalence of episcleritis

The prevalence of episcleritis was assessed in psoriasis patients, divided into subgroups based on the presence or absence of uveitis. In the uveitis subgroup, the proportion of episcleritis was 0.05 (95% CI: 0.02, 0.11; Supplementary Material Fig. 7). For patients without uveitis, the proportion was lower at 0.02 (95% CI: 0.01, 0.03; Supplementary Material Fig. 7). The analysis showed low heterogeneity (*P* = 0.36, I² = 8%).

### Prevalence of Glaucoma

The prevalence of glaucoma was evaluated in psoriasis patients. The overall proportion of glaucoma was low (Proportion = 0.02; 95% CI: 0.02, 0.03; Supplementary Material Fig. 8). Moderate heterogeneity was observed (*P* = 0.03, I² = 67%).

### Prevalence of Iritis

The prevalence of iritis was analyzed in psoriasis patients. The overall proportion of iritis was relatively low (Proportion = 0.03; 95% CI: 0.01, 0.06; Supplementary Material Fig. 9). Moderate heterogeneity was present (*P* = 0.07, I² = 71%).

### Prevalence of keratitis

The prevalence of keratitis was evaluated in psoriasis patients. The overall proportion of keratitis was moderate (Proportion = 0.23; 95% CI: 0.17, 0.29; Supplementary Material Fig. 10). High heterogeneity was observed (*P* < 0.01, I² = 81%).

### Prevalence of lid margin abnormalities

The prevalence of lid margin abnormalities was assessed in psoriasis patients. The overall proportion of lid margin abnormalities was moderate (Proportion = 0.12; 95% CI: 0.07, 0.17; Supplementary Material Fig. 11). High heterogeneity was noted (*P* < 0.01, I² = 88%).

### Prevalence of low visual acuity

The prevalence of low visual acuity was analyzed in psoriasis patients, grouped by the presence or absence of uveitis. Low visual acuity was low in the subgroup without uveitis (Proportion = 0.05; 95% CI: 0.02, 0.08; Supplementary Material Fig. 12). In patients with uveitis, the proportion was significantly higher (Proportion = 0.30; 95% CI: 0.00, 0.78; Supplementary Material Fig. 12). High heterogeneity was observed (*P* < 0.01, I² = 92%).

### Prevalence of macular edema

The prevalence of macular edema was evaluated in psoriasis patients, stratified by the presence of uveitis. In the uveitis subgroup, macular edema was higher (Proportion = 0.11; 95% CI: 0.06, 0.15; Supplementary Material Fig. 13). For patients without uveitis, the proportion was significantly lower (Proportion = 0.01; 95% CI: 0.00, 0.03; Supplementary Material Fig. 13). High heterogeneity was observed (*P* < 0.01, I² = 78%).

### Prevalence of meibomian gland dysfunction

The prevalence of meibomian gland dysfunction was assessed in psoriasis patients. The overall proportion of meibomian gland dysfunction was moderate to high (Proportion = 0.40; 95% CI: 0.34, 0.45; Supplementary Material Fig. 14). High heterogeneity was observed (*P* < 0.01, I² = 95%).

### Prevalence of pinguecula

The prevalence of pinguecula was evaluated in psoriasis patients. The overall proportion of pinguecula was low (Proportion = 0.09; 95% CI: 0.06, 0.11; Supplementary Material Fig. 15). Moderate heterogeneity was noted (*P* = 0.13, I² = 42%).

### Prevalence of posterior synechiae

The prevalence of posterior synechiae was evaluated in psoriasis patients, categorized by the presence of uveitis. High heterogeneity was observed (*P* < 0.01, I² = 96%).

### Prevalence of pseudophakia

The prevalence of pseudophakia was analyzed in psoriasis patients. The overall proportion of pseudophakia was low (Proportion = 0.07; 95% CI: 0.04, 0.11; Supplementary Material Fig. 16). High heterogeneity was noted (*P* < 0.01, I² = 96%).

#### Prevalence of pterygium

The prevalence of pterygium was evaluated in psoriasis patients. The overall proportion of pterygium was low (Proportion = 0.05; 95% CI: 0.03, 0.07; Supplementary Material Fig. 17). Moderate heterogeneity was observed (*P* < 0.01, I² = 75%).

### Prevalence of retinal detachment

The prevalence of retinal detachment was evaluated in psoriasis patients, categorized by the presence of uveitis. In the subgroup without uveitis, the proportion of retinal detachment was 0.00 (95% CI: 0.00, 0.00; Supplementary Material Fig. 18). For patients with uveitis, the proportion was slightly higher at 0.06 (95% CI: 0.01, 0.16; Supplementary Material Fig. 18). Moderate heterogeneity was observed (*P* = 0.09, I² = 66%).

#### Prevalence of retinopathy

The prevalence of retinopathy was assessed in psoriasis patients, divided into subgroups based on the diagnosis of uveitis. In the subgroup without uveitis, the proportion of retinopathy was very low (Proportion = 0.01; 95% CI: 0.00, 0.01; Supplementary Material Fig. 19). For patients with uveitis, the proportion was higher at 0.17 (95% CI: 0.00, 0.64; Supplementary Material Fig. 19). Low heterogeneity was noted (*P* = 0.29, I² = 11%).

#### Prevalence of trichiasis

The prevalence of trichiasis was assessed in psoriasis patients, categorized by the presence of uveitis. Trichiasis was very low in the subgroup without uveitis (Proportion = 0.02; 95% CI: 0.00, 0.04; Supplementary Material Fig. 20). For patients with uveitis, the proportion was significantly higher (Proportion = 0.22; 95% CI: 0.21, 0.24; Supplementary Material Fig. 20). High heterogeneity was observed (*P* = 0, I² = 100%).

#### Prevalence of uveitis

The incidence of uveitis was evaluated in psoriasis patients, stratified by the presence of previous uveitis. In patients without previous uveitis, the proportion of uveitis was very low (Proportion = 0.01; 95% CI: 0.00, 0.29; Supplementary Material Fig. 21). For patients with previous uveitis, the proportion was significantly higher (Proportion = 0.36; 95% CI: 0.12, 0.61; Supplementary Material Fig. 21). High heterogeneity was noted (*P* < 0.01, I² = 100%).

### Prevalence of vitritis

The prevalence of vitritis was evaluated in psoriasis patients, stratified by the presence of uveitis. In the uveitis subgroup, vitritis was moderate (Proportion = 0.28; 95% CI: 0.00, 0.82; Supplementary Material Fig. 22). For patients without uveitis, the proportion was very low (Proportion = 0.01; 95% CI: 0.00, 0.01; Supplementary Material Fig. 22). High heterogeneity was noted (*P* < 0.01, I² = 99%).

### Control population versus psoriatic patients relative likelihood analysis

#### Odds of blepharitis

Compared to the control group, the odds ratio of developing blepharitis in psoriasis patients was 1.46 (95% CI: 0.94, 2.28; Supplementary Material Fig. 23). This result indicates no significant difference between the two groups. High heterogeneity was observed among the studies (*P* < 0.01, I² = 89%).

### Odds of cataracts

The odds ratio of developing cataracts in psoriasis patients compared to the control group was 1.27 (95% CI: 0.79, 2.04; Supplementary Material Fig. 24). This result indicates no significant difference between the two groups. Additionally, no significant heterogeneity was observed among the studies (*P* = 0.47, I² = 0%).

### Odds of conjunctival hyperemia

**Fig. 2:**
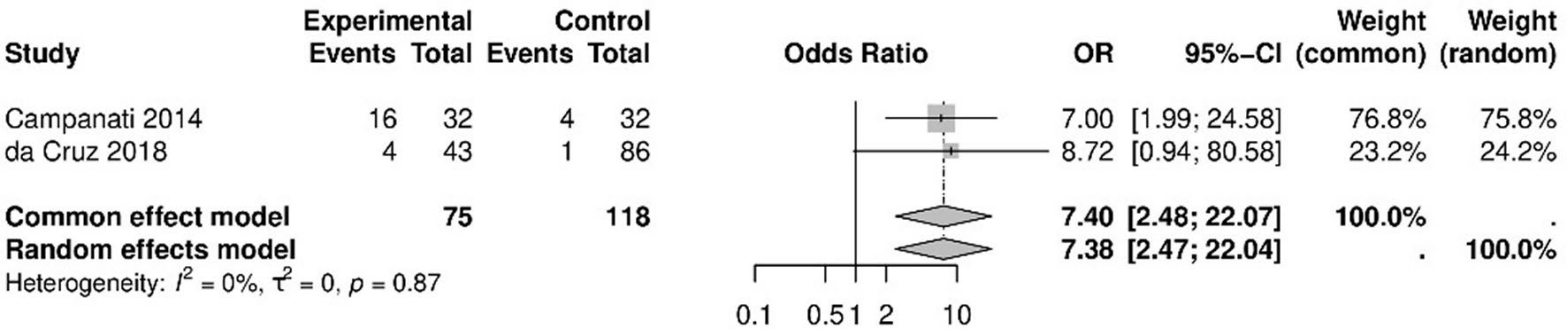
Conjunctival hyperemia forrest plot

The odds ratio of developing conjunctival hyperemia in psoriasis patients compared to the control group was 7.38 (95% CI: 2.47, 22.04; Fig. 2). This indicates a significant difference between the two groups. No significant heterogeneity was observed among the studies (*P* = 0.87, I² = 0%).

#### Odds of conjunctivitis

**Fig. 3:**
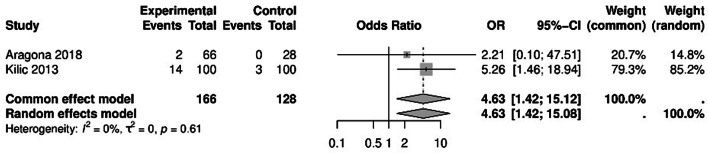
Conjunctivitis dry eye

The odds ratio of developing conjunctivitis in psoriasis patients compared to the control group was 4.63 (95% CI: 1.42, 15.08; Fig. 3). This indicates a significant difference between the two groups. No significant heterogeneity was observed among the studies (*P* = 0.61, I² = 0%).

#### Odds of dry eye

**Fig. 4:**
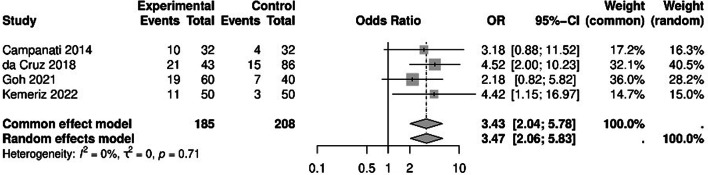
Dry eye forrest plot

The odds ratio of developing dry eye in psoriasis patients compared to the control group was 3.47 (95% CI: 2.06, 5.83; Fig. 4). This indicates a significant difference between the two groups. No significant heterogeneity was observed among the studies (*P* = 0.71, I² = 0%) Fig. 4.

## Odds of episcleritis

The odds ratio of developing episcleritis in psoriasis patients compared to the control group was 0.77 (95% CI: 0.20, 3.00; Supplementary Material Fig. 25). This indicates no significant difference between the two groups. No significant heterogeneity was observed among the studies (*P* = 0.35, I² = 0%).

## Odds of Glaucoma

Compared to the control group, the odds ratio of developing glaucoma in psoriasis patients was 1.00 (95% CI: 0.88, 1.14; Supplementary Material Fig. 26). This indicates no significant difference between the two groups. No significant heterogeneity was observed among the studies (*P* = 0.82, I² = 0%).

### Odds of meibomian gland dysfunction

**Fig. 5:**
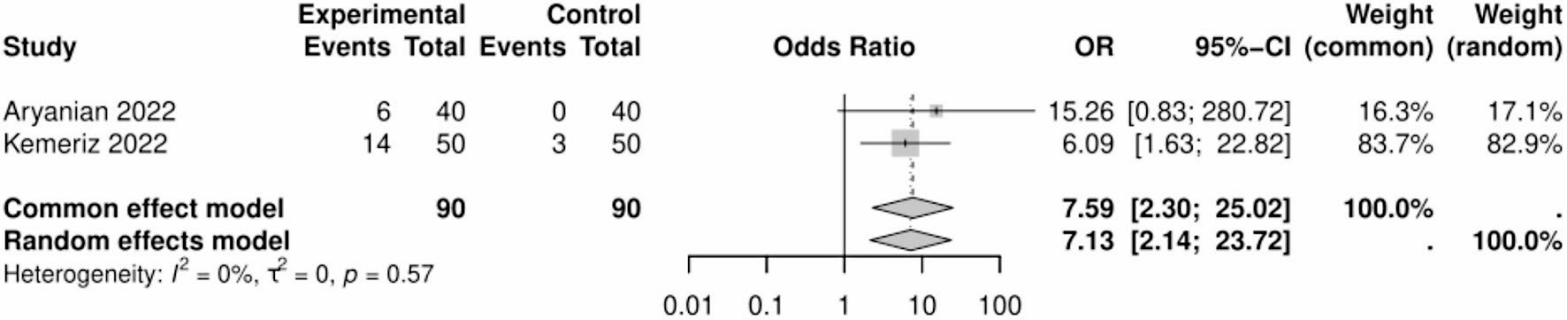
Meibomian gland dysfunction forrest plot

The odds ratio of developing meibomian gland dysfunction in psoriasis patients compared to the control group was 7.13 (95% CI: 2.14, 23.72; Fig. 5). This indicates a significant difference between the two groups. No significant heterogeneity was observed among the studies (*P* = 0.57, I² = 0%).

#### Odds of pinguecula

The odds ratio of developing pinguecula in psoriasis patients compared to the control group was 0.81 (95% CI: 0.43, 1.52; Supplementary Material Fig. 28). This indicates no significant difference between the two groups. No significant heterogeneity was observed among the studies (*P* = 0.87, I² = 0%).

### Odds of pterygium

The odds ratio of developing pterygium in psoriasis patients compared to the control group was 1.58 (95% CI: 0.77, 3.25; Supplementary Material Fig. 29). This indicates no significant difference between the two groups. No significant heterogeneity was observed among the studies (*P* = 0.83, I² = 0%).

#### Odds of uveitis

The odds ratio of developing uveitis in psoriasis patients compared to the control group was 1.03 (95% CI: 0.74, 1.43; Supplementary Material Fig. 30). This indicates no significant difference between the two groups. No significant heterogeneity was observed among the studies (*P* = 0.74, I² = 0%).

## Discussion

In our systematic review and meta-analysis, which included 30 studies with 131,687 patients, we performed a single-arm analysis to investigate the prevalence of 24 ocular diseases in patients diagnosed with psoriasis. Whenever data was available, we also compared the prevalence of these diseases in patients with a uveitis diagnosis or a previous uveitis diagnosis in the case of uveitis analysis. Additionally, we analyzed the relative likelihood of 9 ocular pathologies in patients with psoriasis compared to those without psoriasis.

In our single-arm analysis, we calculated the overall prevalence of ocular diseases in patients with psoriasis and examined the impact of associated or previous uveitis on this prevalence. When comparing the prevalence of diseases with associated uveitis, there was a significant increase in the following diseases: Cataracts, Low Visual Acuity, Macular Edema, Retinal Detachment, Trichiasis, Vitritis, and Uveitis. Conversely, there was a significant decrease in the prevalence of Blepharitis and Dry Eye in patients with associated uveitis. No statistically significant difference was observed between the non-uveitis and uveitis groups in the following diseases: Corneal Opacity, Elevated Intraocular Pressure, Episcleritis, and Retinopathy.

Our analysis comparing the relative likelihood of psoriatic patients and non-psoriatic control population revealed as it follows: Blepharitis, Cataracts, Episcleritis, Glaucoma, Pinguecula, Pterygium, and Uveitis did not differ significantly between psoriasis patients and control patients. In contrast, the following conditions showed a statistically significant higher relative likelihood of developing in psoriasis patients compared to the control population: Conjunctival Hyperemia, Conjunctivitis, Dry Eye, and Meibomian Gland Dysfunction.

Psoriasis patients exhibit a significantly faster tear-film break-up time and a higher rate of epithelial cells undergoing metaplasia [13, 37, 38]. Additionally, patients with psoriasis have been shown to have significant tear hyperosmolarity, contributing to tear film malfunction and qualitative dysfunction of the meibomian glands, producing the lipid component of tears. Patients with psoriasis also have higher gland plugging rates and abnormal thickness of meibomian gland secretion [37, 39]. The well-documented association between psoriasis pathophysiology and tear malfunction, along with meibomian gland dysfunction, is reflected in our findings of an increased relative likelihood of Dry Eye, Meibomian Gland Dysfunction, Conjunctivitis, and Conjunctival hyperemia in the psoriatic population compared to the control population.

Our meta-analysis indicates that the relative likelihood of uveitis in psoriatic patients is not statistically higher than in the control population. However, when present, uveitis can lead to severe sequelae that are visually devastating, such as elevated intraocular pressure, optic neuropathy, macular edema, retinal vasculitis, and permanent visual loss [40]. Due to the severe ocular inflammation caused by uveitis, this pathology is often accompanied by other ophthalmological diseases [41]. Our analysis shows an increased prevalence of cataracts, low visual acuity, macular edema, retinal detachment, trichiasis, vitritis, and uveitis in psoriatic patients with active or previous uveitis. Conversely, uveitis is a protective factor against blepharitis and dry eye in patients with psoriasis. Previous studies have reported an increased risk of uveitis in severe psoriasis [42]. Additionally, a recent study by Oliveira et al. showed that severe psoriasis, indicated by a Psoriasis Area and Severity Index (PASI) score > 10, is a protective factor for dry eye [23]. The mechanisms behind these findings are not yet fully understood; however, severe psoriasis, represented by psoriasis with uveitis, appears to be a protective factor against blepharitis and dry eye.

Our meta-analysis had limitations and biases due to the quality of the selected studies. The results of this meta-analysis provide valuable insights into the prevalence of ocular disease in psoriatic patients and the relative likelihood of developing certain ocular diseases when compared to the non-psoriatic population. However, it is essential to consider the limitations of this study. Although including 30 studies is a strength, a larger dataset would provide more robust and generalizable conclusions. High heterogeneity in some outcomes suggests variability in prevalence across studies. This variability could be influenced by factors such as variations in patient populations, disease severity, and study methodologies. The possibility of publication bias cannot be ruled out, as studies with positive or significant results may be more likely to be published. In contrast, studies with negative or non-significant findings may not. This bias may have affected the overall conclusions of the meta-analysis. The meta-analysis relied on aggregated data from published studies. Individual patient data were unavailable for analysis, which could have provided a more detailed understanding of the incidence of ophthalmology conditions in psoriasis. The included studies were conducted in various countries, potentially introducing patient demographics and follow-up protocol variability. This may limit the generalizability of the findings to a broader population. Additionally, as this meta-analysis includes observational studies, the influence of confounders on the results cannot be excluded.

## Conclusion

In conclusion, this systematic review and meta-analysis demonstrate the prevalence of 25 ocular diseases in psoriatic patients. Also, it shows that psoriasis patients have an increased relative likelihood of developing conjunctival hyperemia, conjunctivitis, dry eye, and meibomian gland dysfunction. Additionally, psoriatic patients who present or have a history of uveitis or have an increased prevalence of cataracts, low visual acuity, macular edema, retinal detachment, trichiasis, vitritis, uveitis, a decreased risk for blepharitis and dry eye when compared to a non-psoriatic control population. There is a need for more studies on ophthalmological manifestations of psoriasis, especially studies that grade the patients by psoriasis severity.

## Electronic supplementary material

Below is the link to the electronic supplementary material.

Supplementary Material 1

## Data Availability

Research data supporting this publication are available to the corresponding author upon request.

## Abbreviations

TNF: Tumor Necrosis Factor
PsA: Psoriatic Arthritis
PRISMA: Preferred Reporting Items for Systematic Review and Meta-Analysis
RR: Relative Risk
SMD: Standardized Mean Differences
SD: Standard Deviation
CI: Confidence Interva
PASI: Psoriasis Area and Severity Index
