# Supplementary material for: Ocular complications in psoriatic patients: a systematic review and meta-analysis
Source: J Ophthalmic Inflamm Infect. 2025 Mar 17;15:29. doi: 10.1186/s12348-025-00486-6 (PMC11914409; doi:10.1186/s12348-025-00486-6)

**Supplementary Material**

**Supplementary Material Methods:**

The following information was extracted whenever available in the articles: First Author Name, Year of Publication, Study design, Number of patients, Number of Control Patients, Psoriatic Patients, Age (Psoriatic Patients), Age (Control Patients), Woman (Psoriatic Patients), Woman (Control Patients), Man (Psoriatic Patients), Man (Control Patients), Metabolic Syndrome, Intervention, Follow-up, Cataract, Dry eyes, Keratoconjunctivitis sicca, Conjunctivitis, Superficial punctate keratitis, Punctate keratitis, Keratitis, Keratic precipitates, Anterior chamber flare, Aqueous cells, Blepharitis, Anterior Blepharitis, Psuedophakia, Corneal opacity, Lens opacity, Vitreous opacity, Dellen of the cornea, Mebomitis, Trichiasis, Uveitis, Posterior synechae, Only blepharoconjunctivitis, Blepharoconjunctivitis with non-specific corneal opacities, Blepharoconjunctivitis with cataract, Blepharoconjunctivitis with non-specific corneal opacities and cataract, Blepharoconjunctivitis with corneal pigment dispersion, Meibomian gland dysfunction (MGD), Meibometry, Meibomian gland occlusion, Mucocutaneous junction shifts, Meibomian gland loss (%), MG distortion, Upper Meiboscore, Lower Meiboscore, Meibomitis, Glaucoma, Intraocular hypertension, Low visual acuity, Scleritis, Episcleritis, Pupillary seclusion, Elevated intraocular pressure, Vitritis, Posterior vitreous detachment (PVD), Vitreous debris, Retinal detachment, Chorioretinal atrophy, Epiretinal membrane, Epimacular membrane, Macular edema, Retinal vasculitis, Central Serous Chorioretinopathy, Hyperpigmentation of the retinal pigment epithelium (RPE), Pigment dispersion, Retinal pigmentary disturbance, Pannus, Iritis, Iridocyclitis, Iris bombe, Iris neovascularization, Retinal hole/tear, Increased cup/disc ratio, Ectropion, Flame hemorrhage at disc, Retinal hemorrhage, Drusen, Tilted disc, Hyperemic disc, Central serous retinopathy, Pituitary adenoma, Pinguecula, Pterygium, Microvascular abnormalities, Vascular leakage, Peripheral venous leakage, Retinal vascular occlusion, Retinopathy, Retinal swelling, Conjunctival hyperemia, Corneal staphyloma, Amblyopia, Lid margin abnormalities, Lid margin irregularity (notching), Myopic refraction, Non-perfusion in the superficial inner retinal capillary vascular plexuses (SCP), Non-perfusion in the deep inner retinal capillary vascular plexuses (DCP), Central foveal thickness (CFT), Foveal vascular density (FD), Foveal avascular zone (FAZ), Subfoveal choroidal thickness (CT), Temporal CT 500 μm, Nasal CT 500 μm, Tear osmolarity, Tear film break-up time, Schirmer test, Corneal staining, Ocular surface disease index, Tarsal conjunctival hyperemia, Tarsal conjunctival papillae, Tarsal conjunctival follicles, Tarsal conjunctival concretions, Lagophthalmos, Early nuclear opalescence, Pseudophakia, Mild nonproliferative diabetic retinopathy, Asteroid hyalosis, Branch retinal vein occlusion, Eyelid malpositions, Bulbar congestion, Bulbar atrophy, Telangiectasia, Fibrous exudates, Koeppe nodules, Busacca nodules, Hypopyon, and Stargardt disease.

**Supplementary Material Figures:**

**Supplementary Material Figure 1**
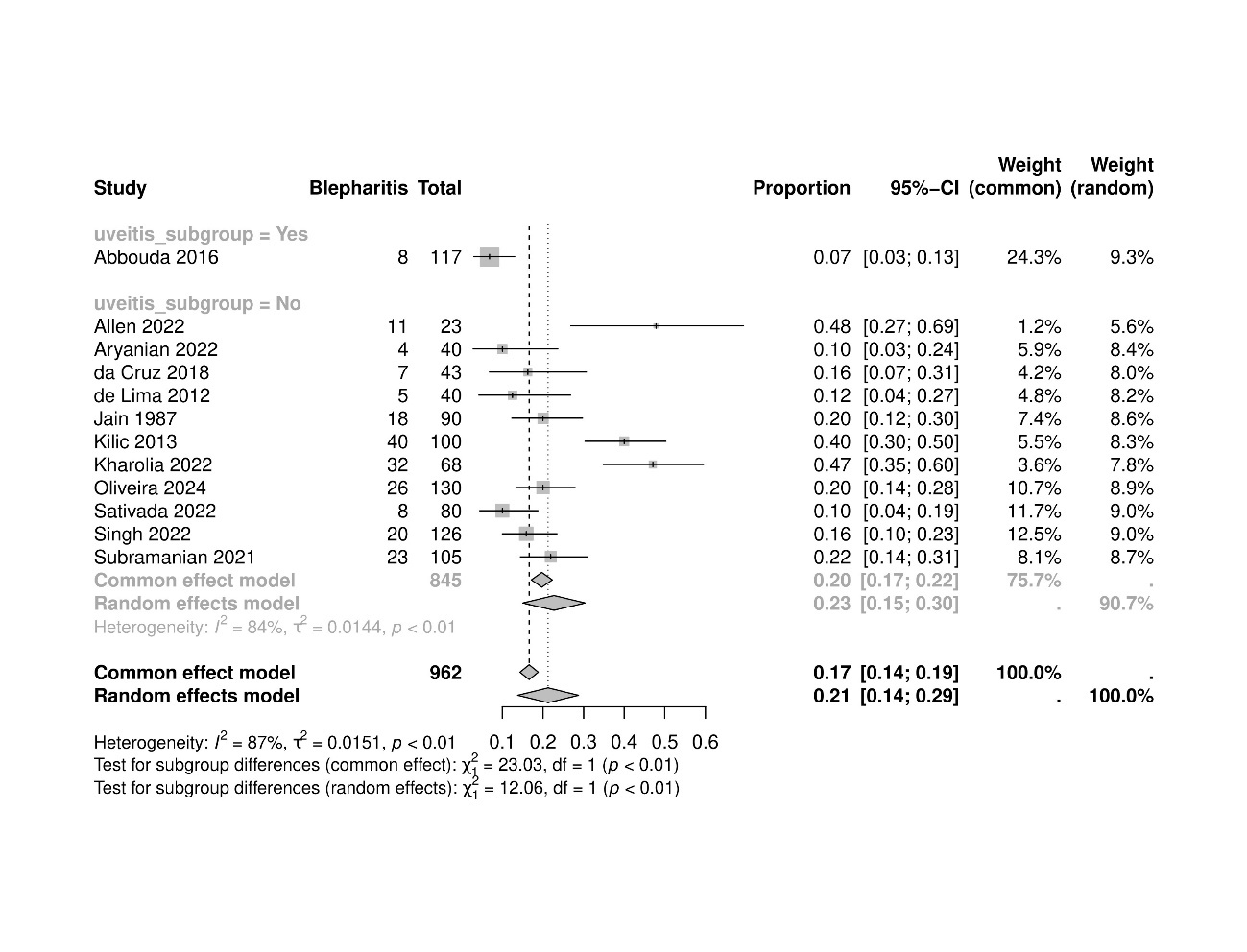


**Supplementary Material Figure 2**
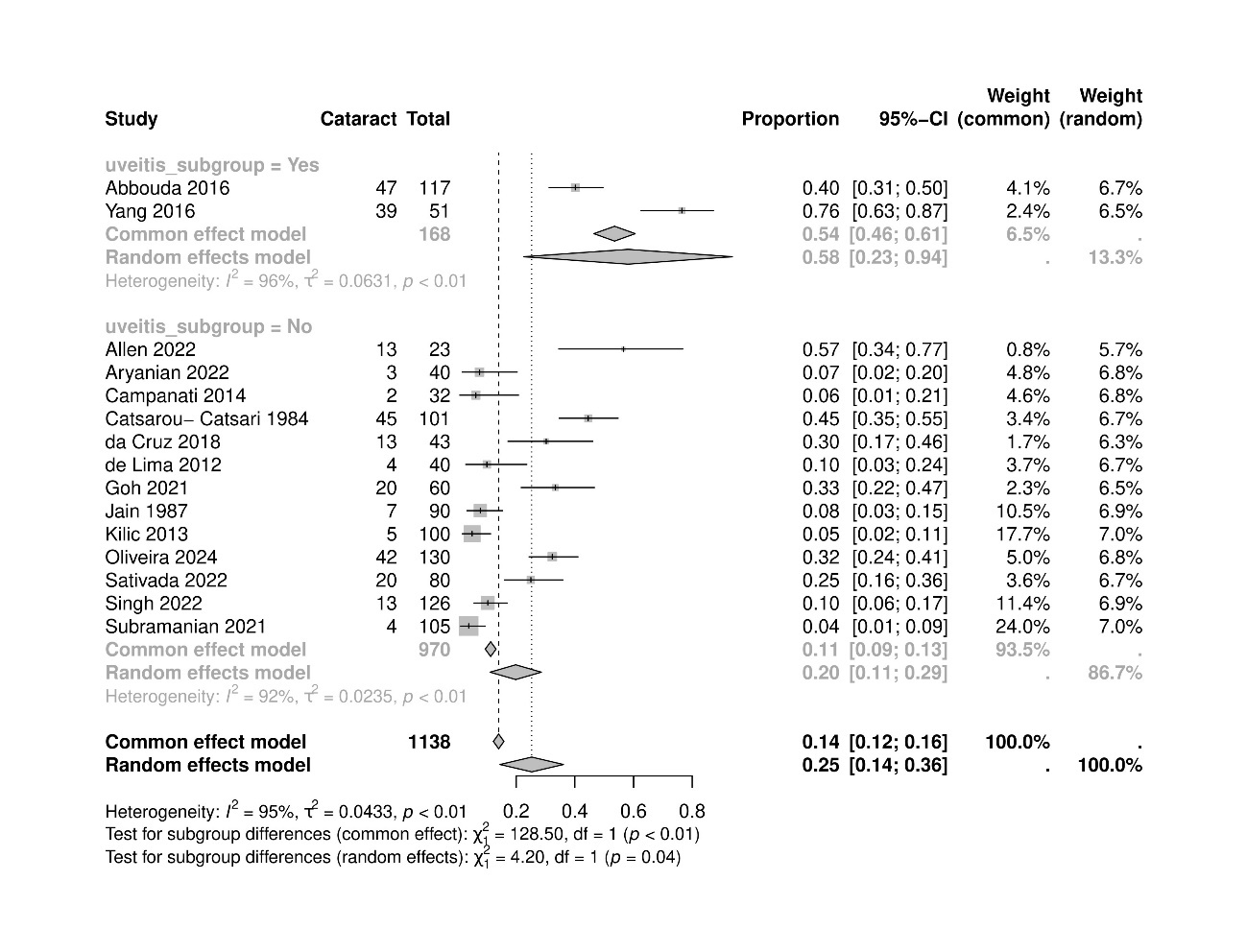


**Supplementary Material Figure 3**
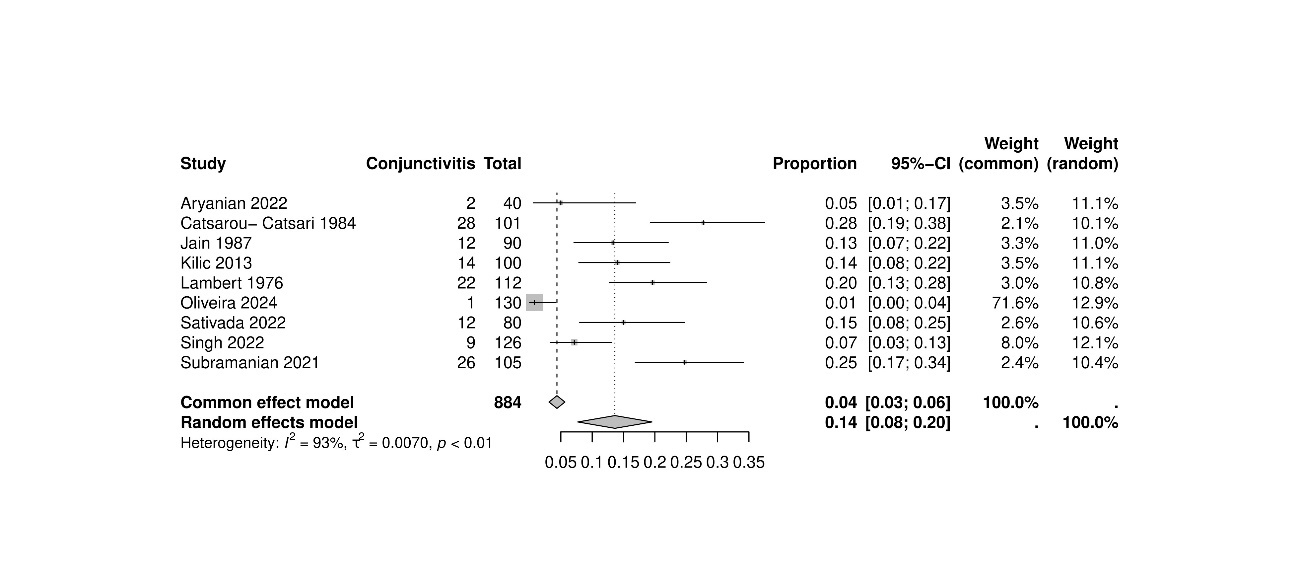


**Supplementary Material Figure 4**
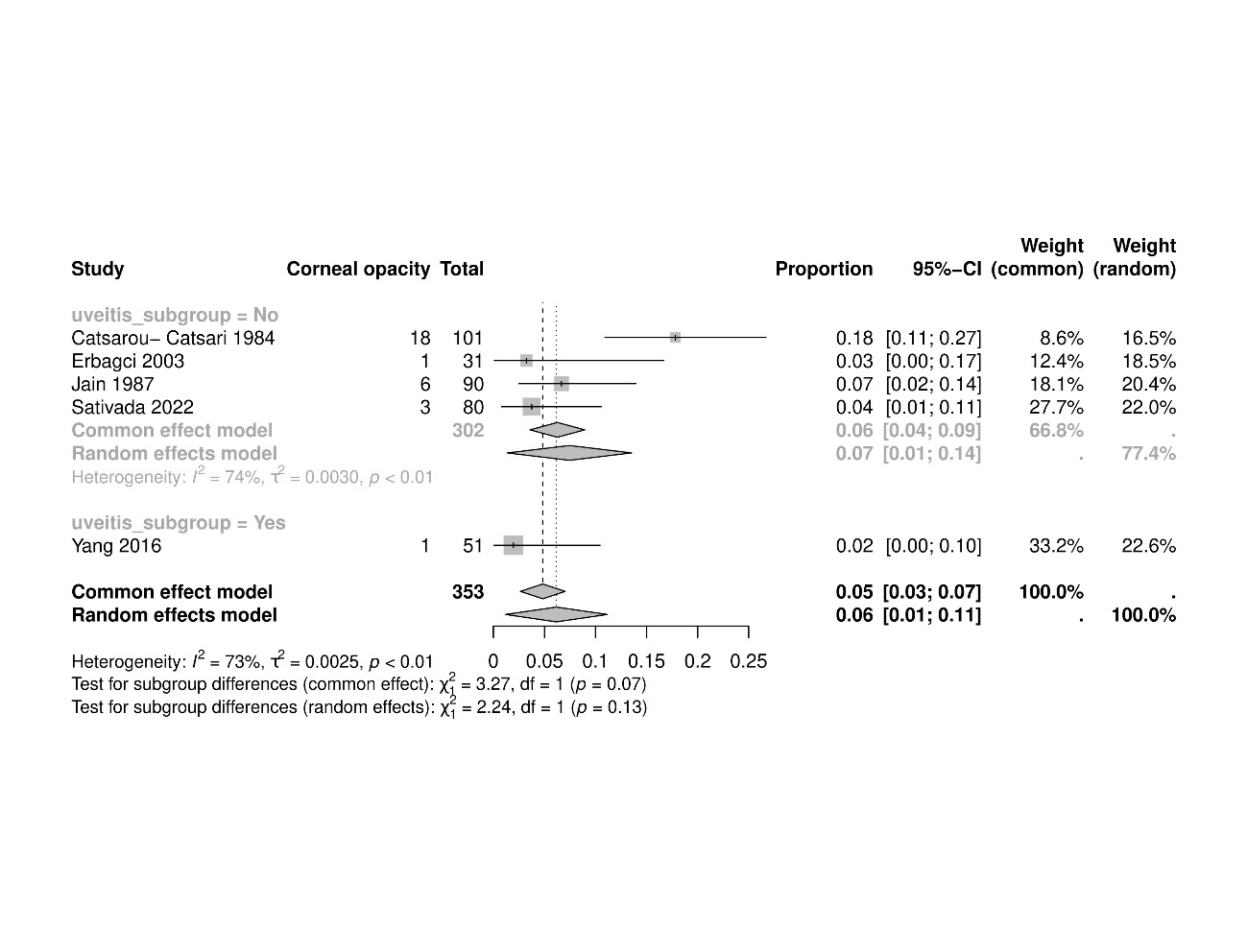


**Supplementary Material Figure 5**
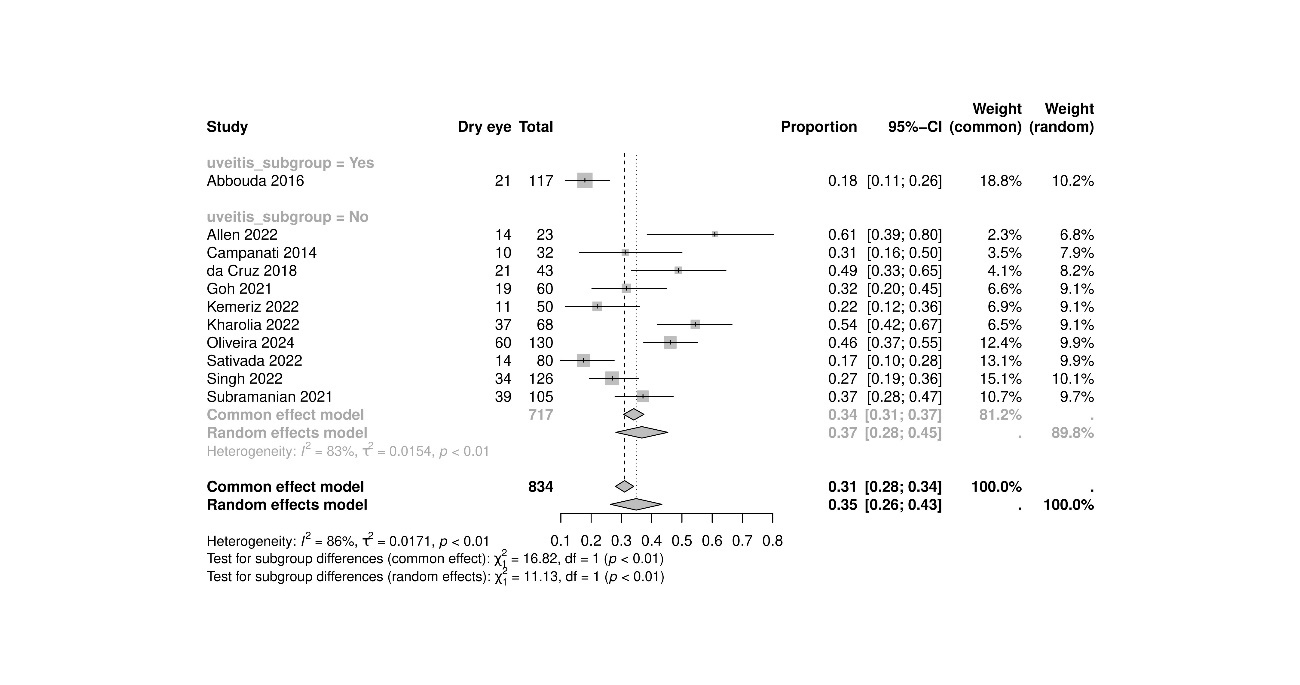


**Supplementary Material Figure 6**
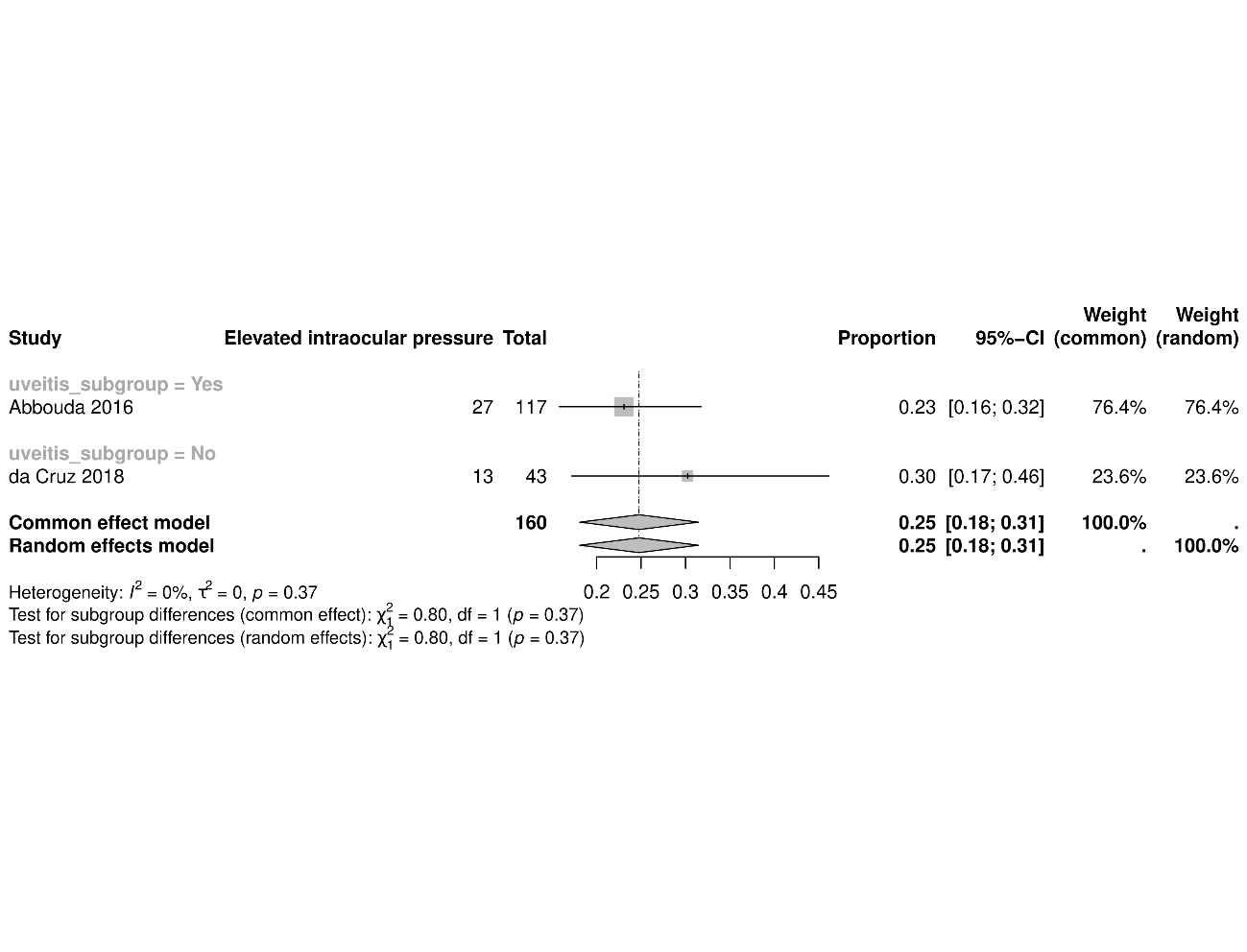


**Supplementary Material Figure 7**
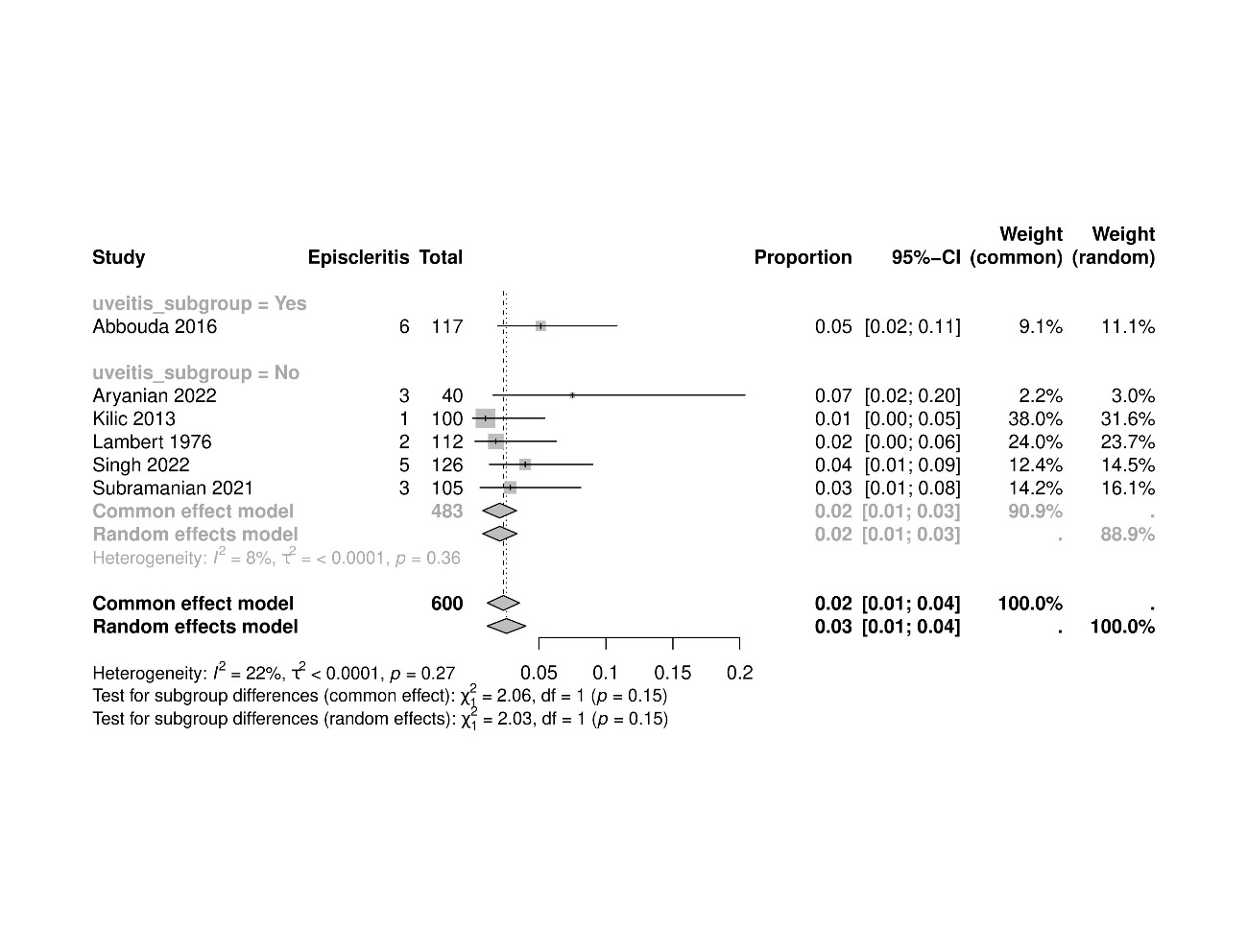


**Supplementary Material Figure 8**
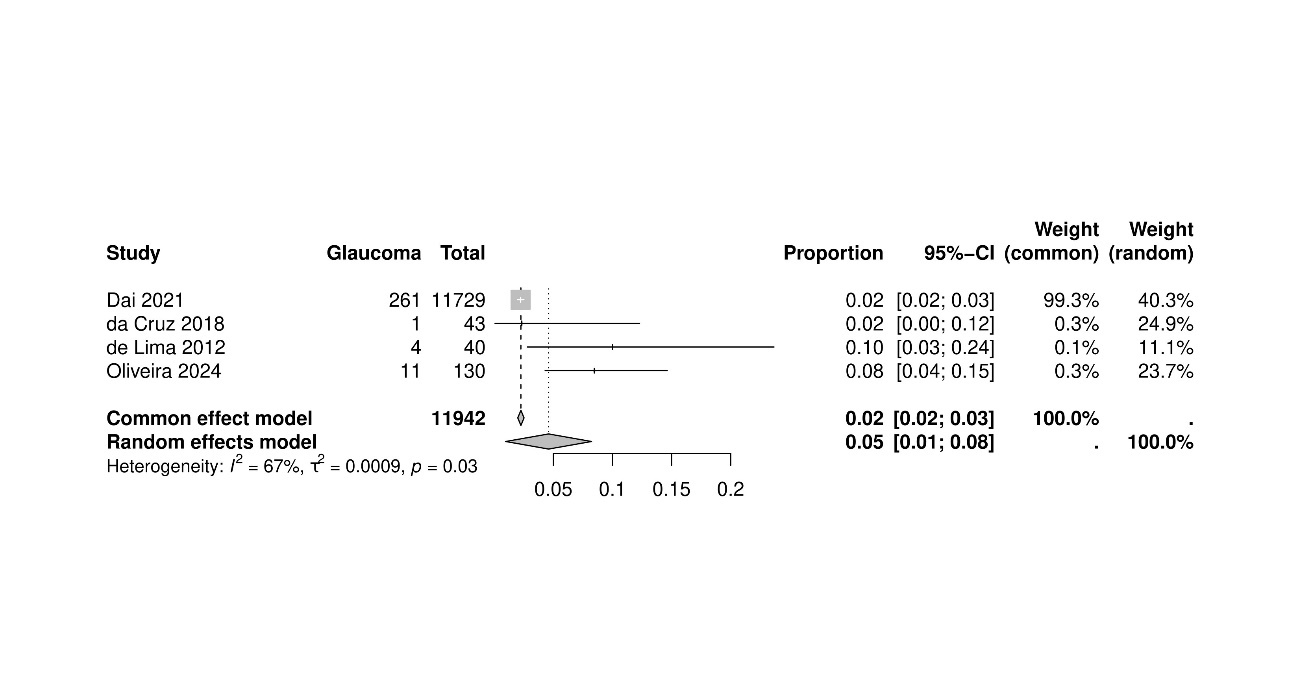


**Supplementary Material Figure 9**
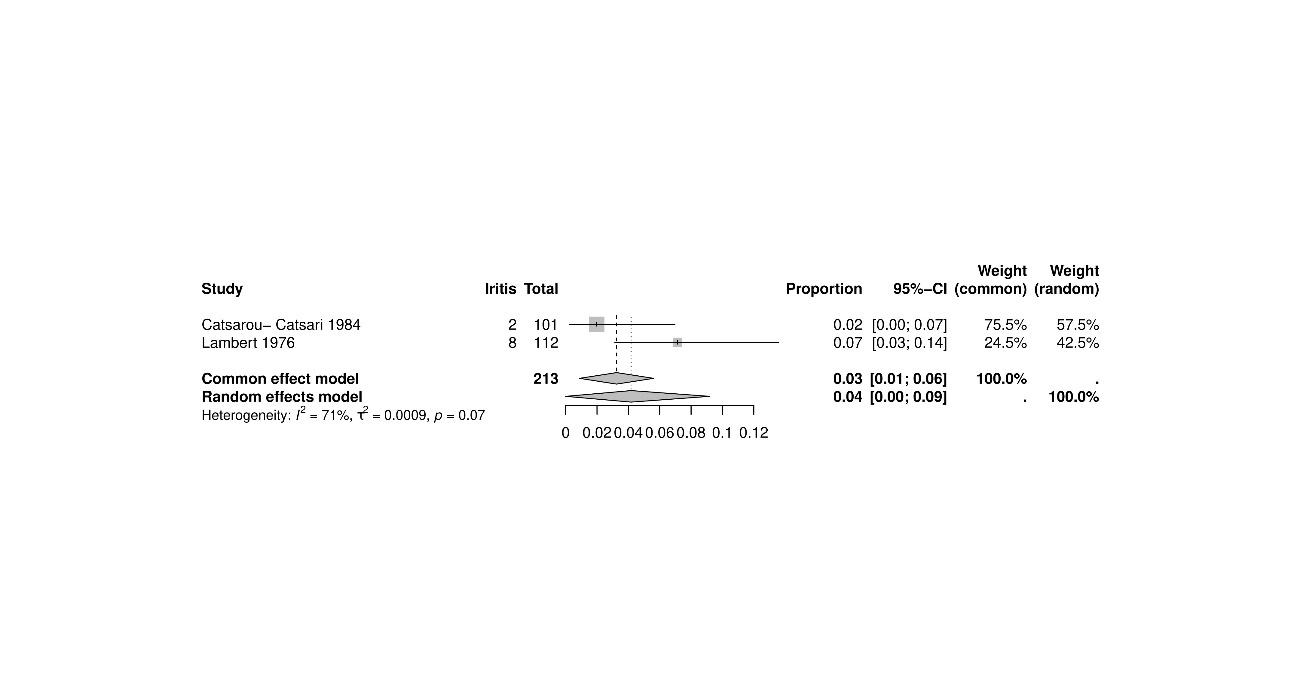


**Supplementary Material Figure 10**
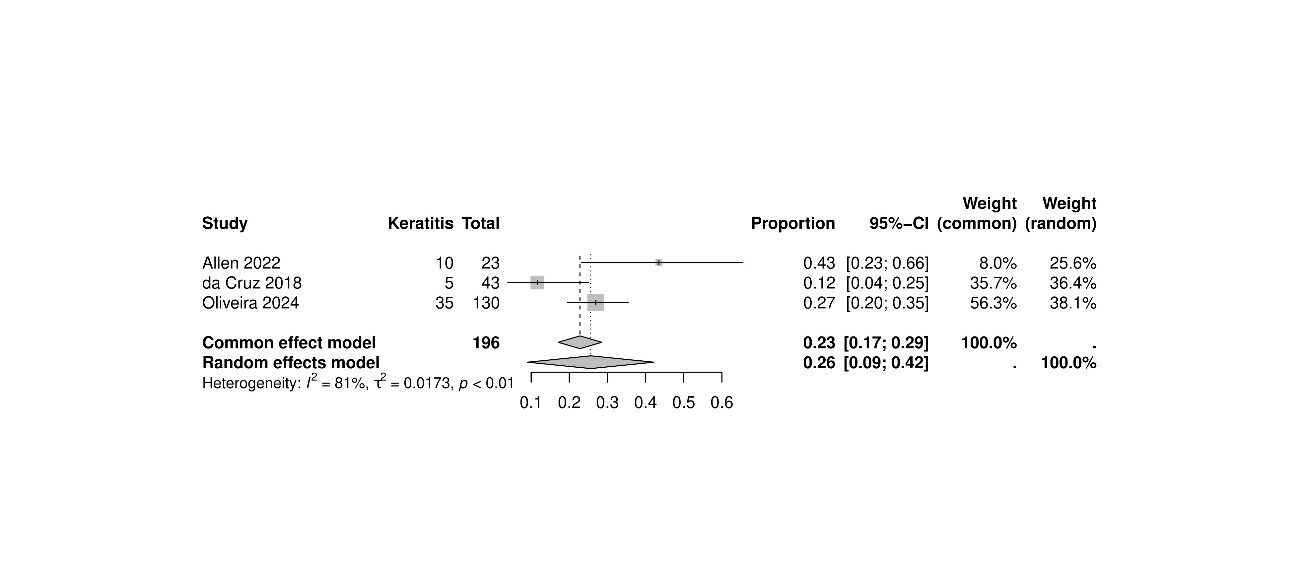


**Supplementary Material Figure 11**
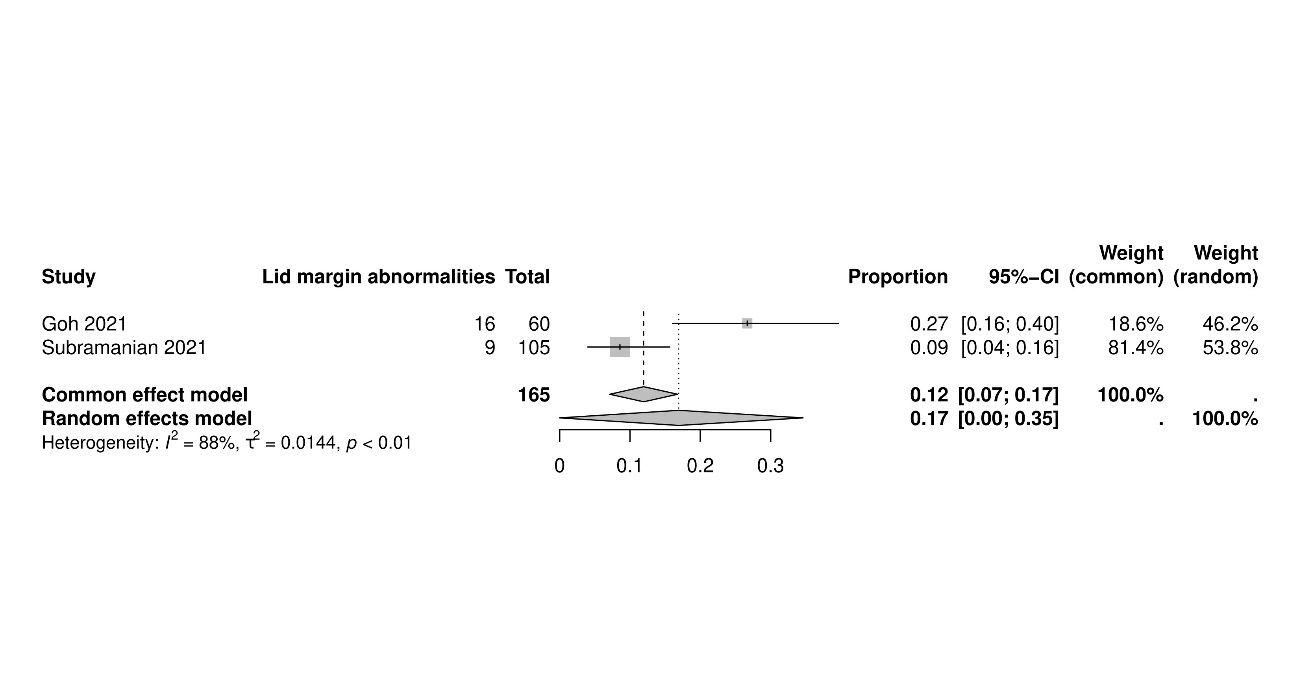


**Supplementary Material Figure 12**


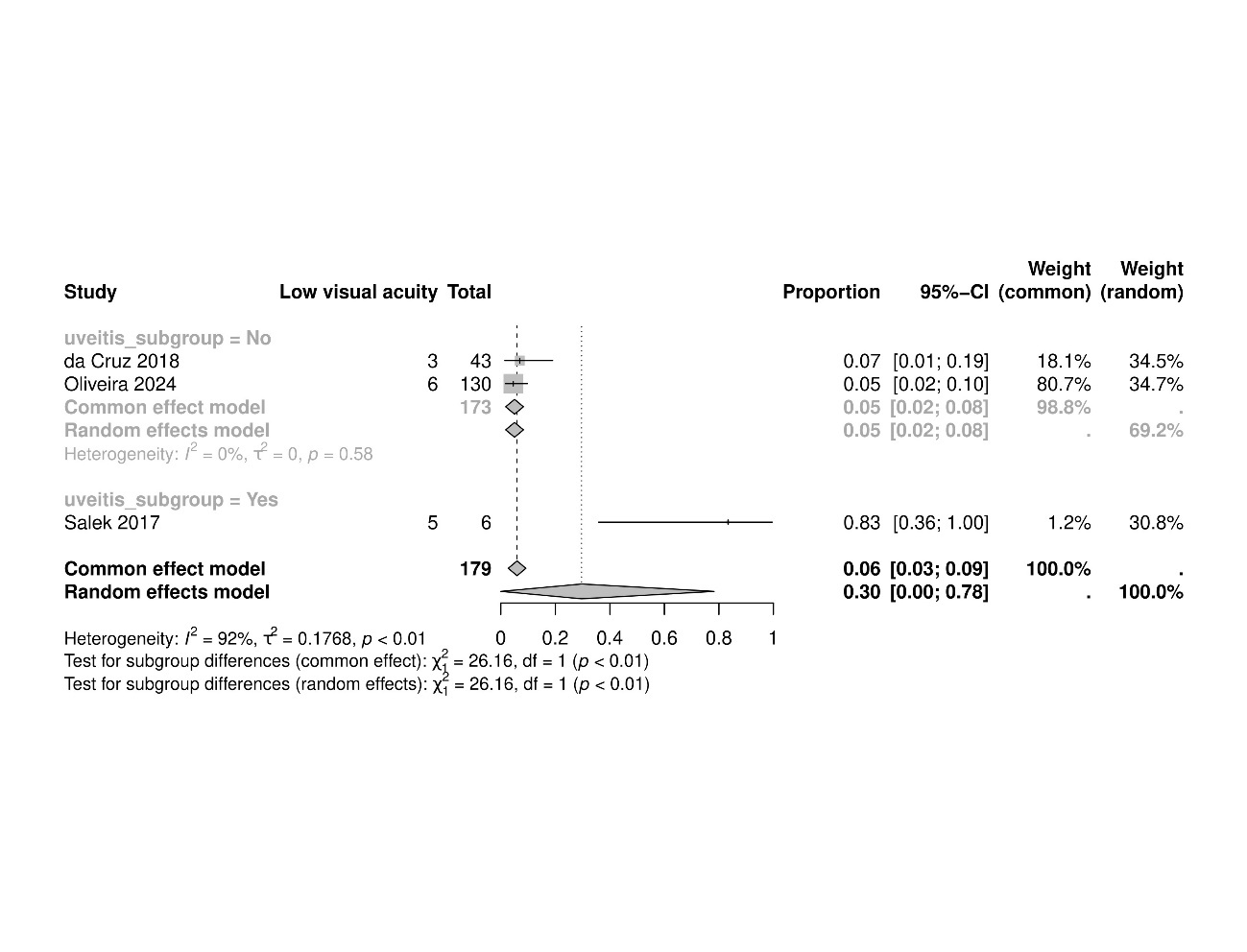


**Supplementary Material Figure 13**
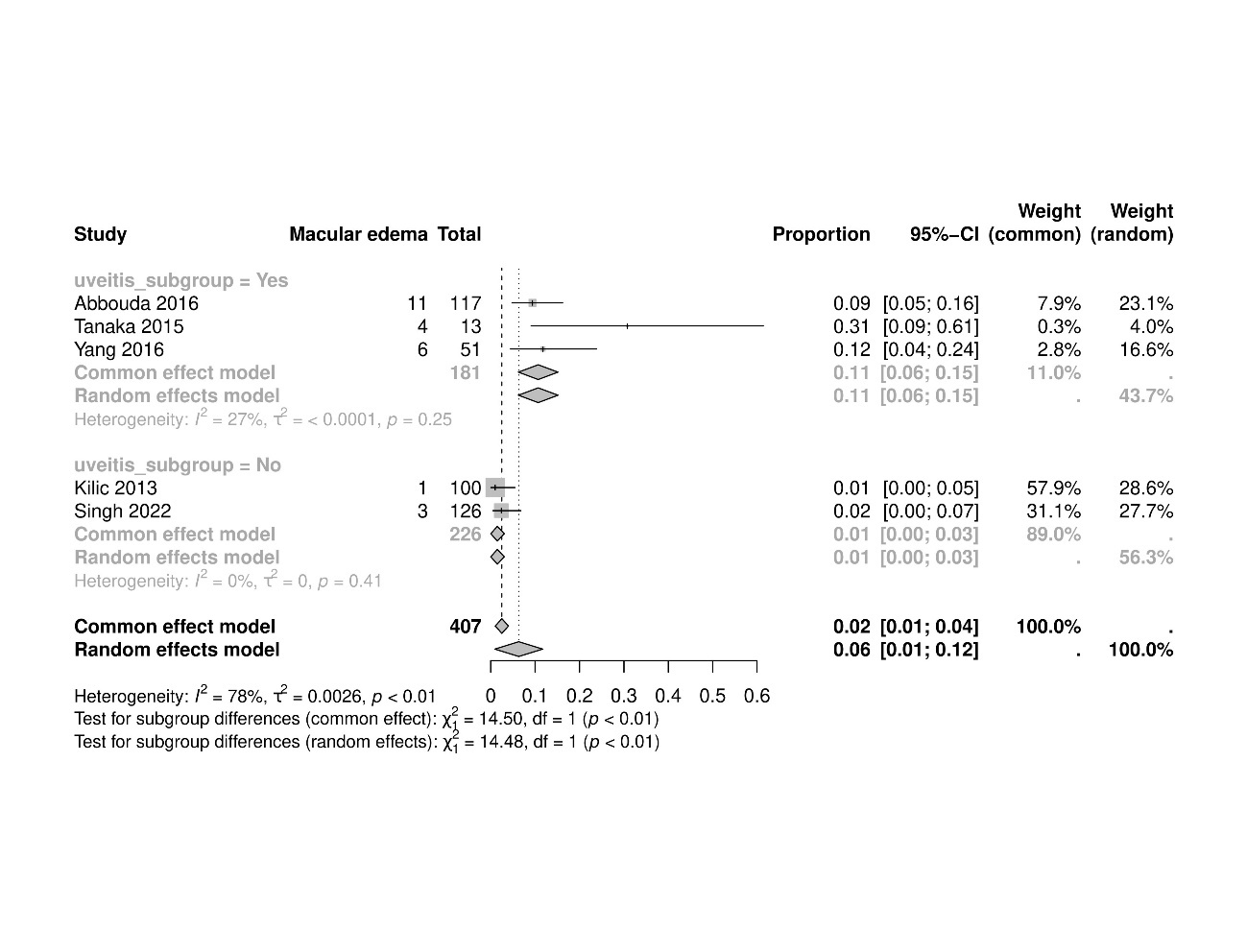


**Supplementary Material Figure 14**
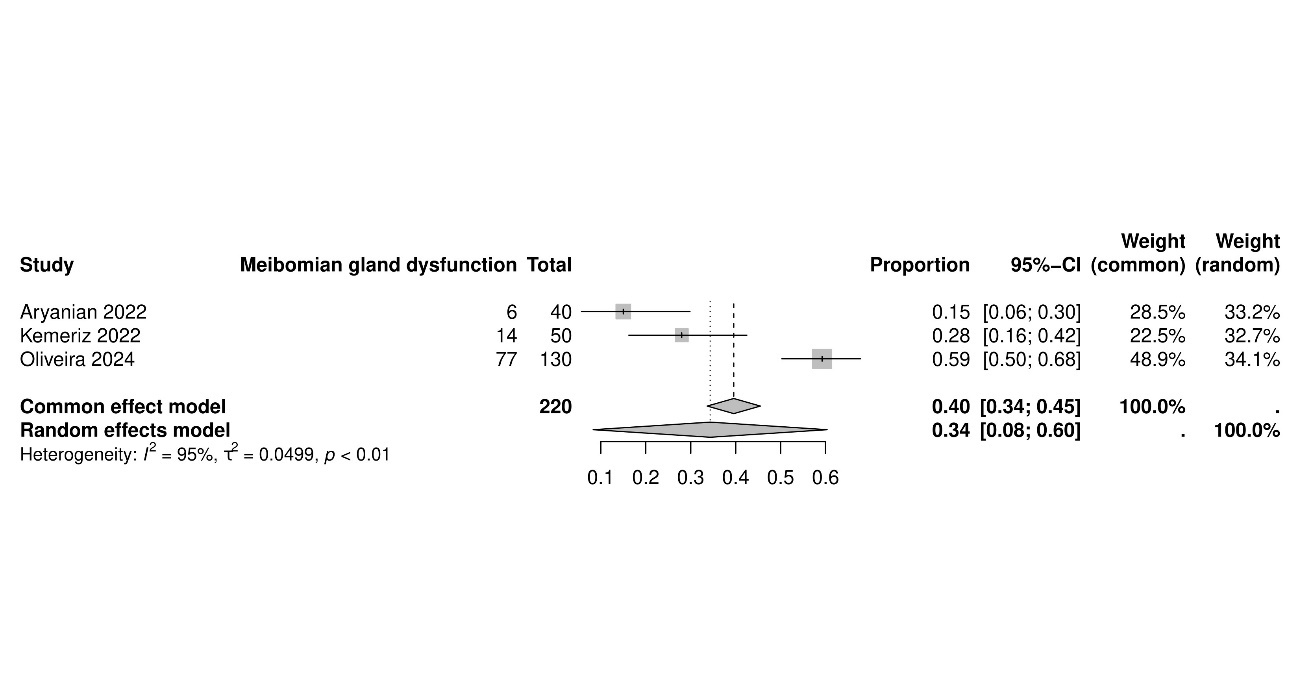


**Supplementary Material Figure 15**
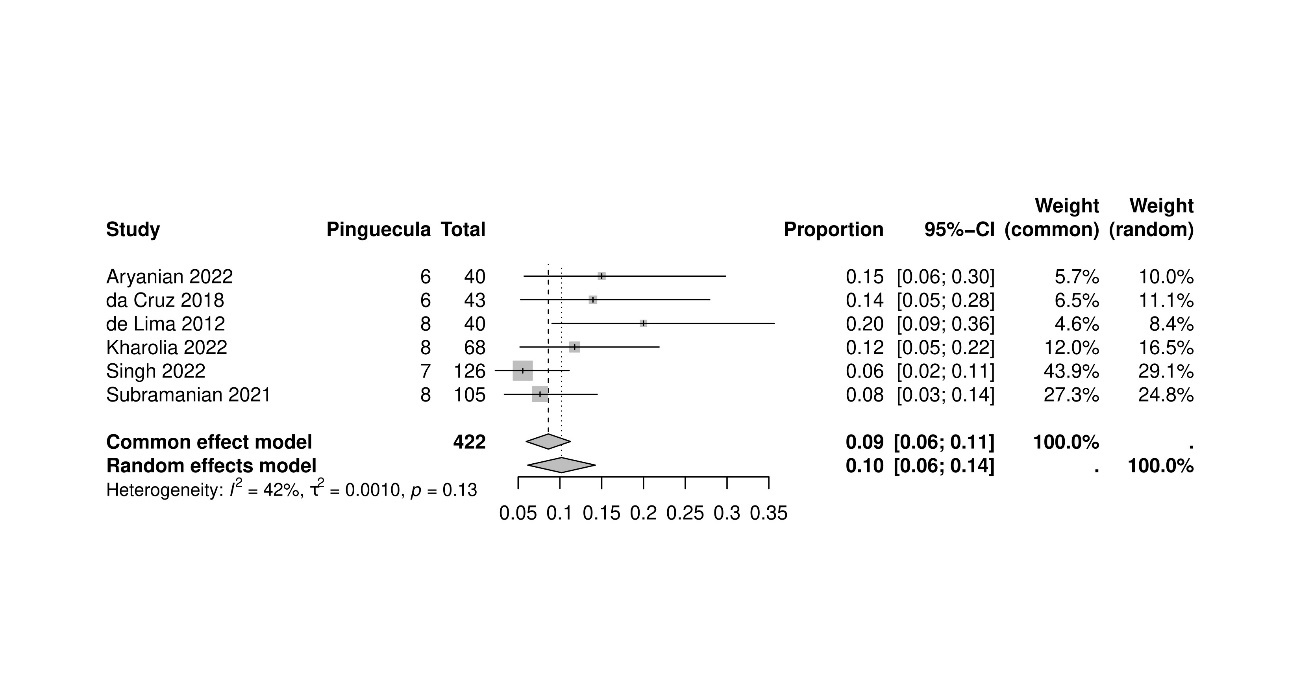


**Supplementary Material Figure 16**


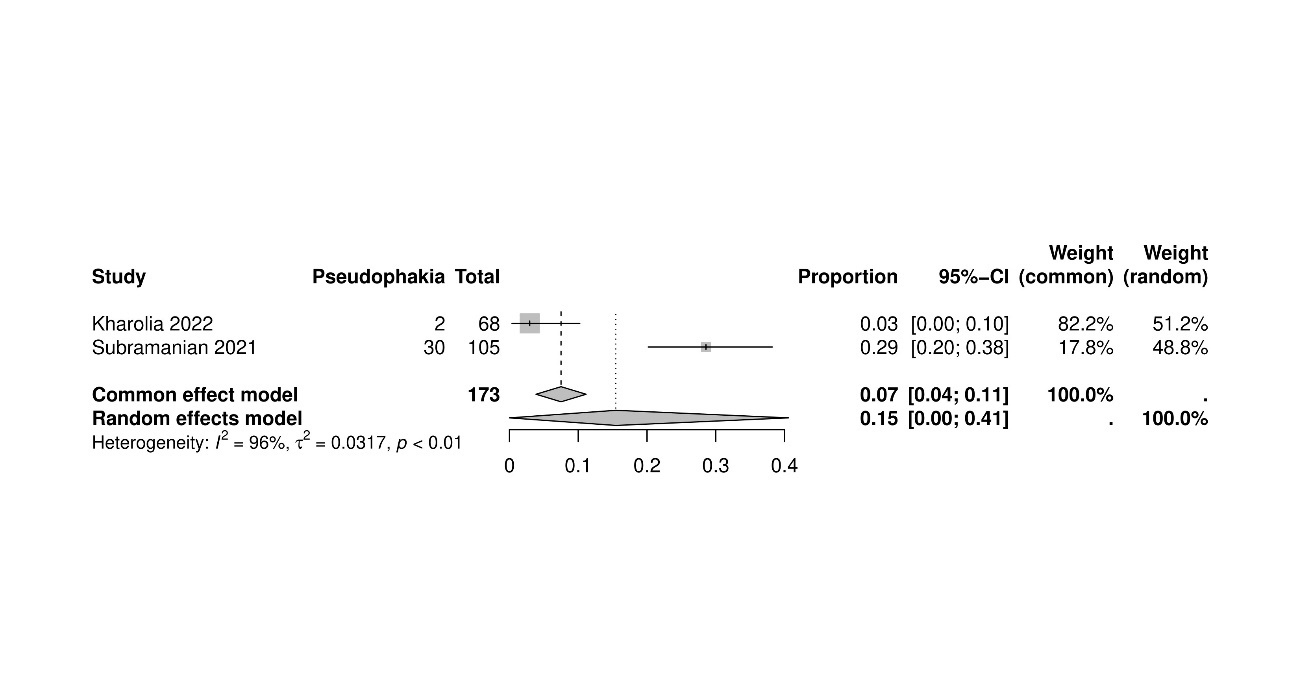


**Supplementary Material Figure 17**
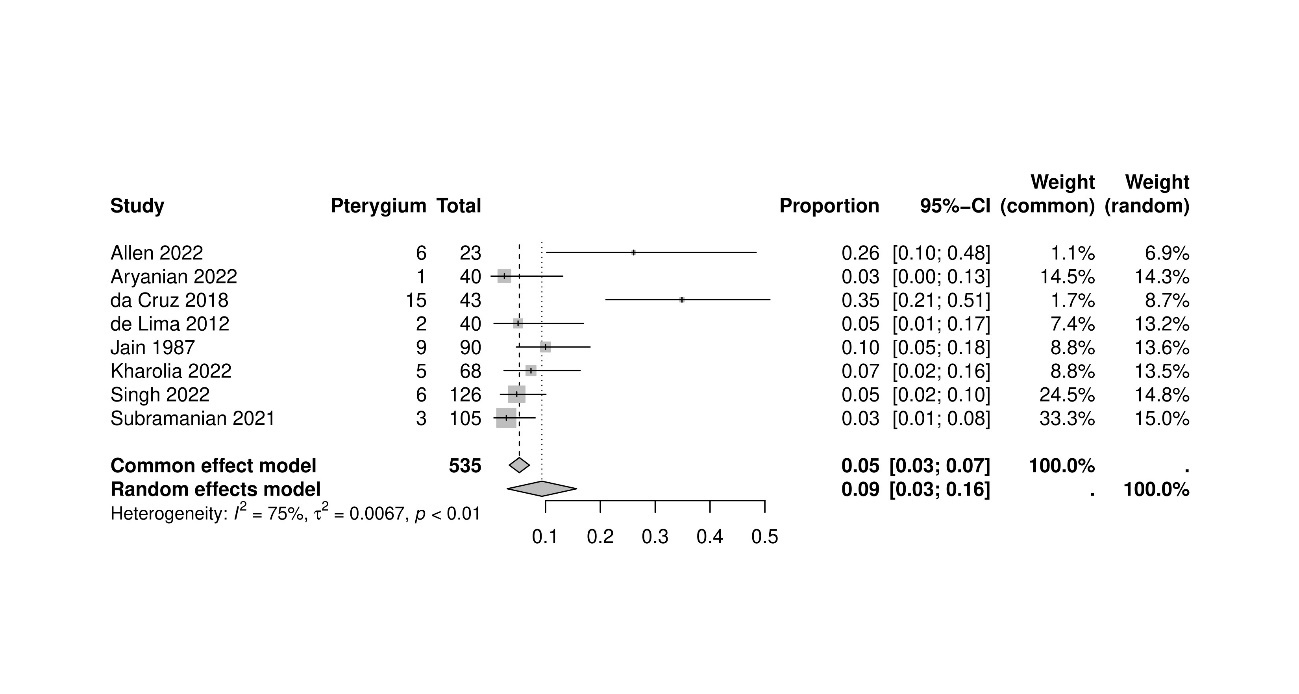


**Supplementary Material Figure 18**
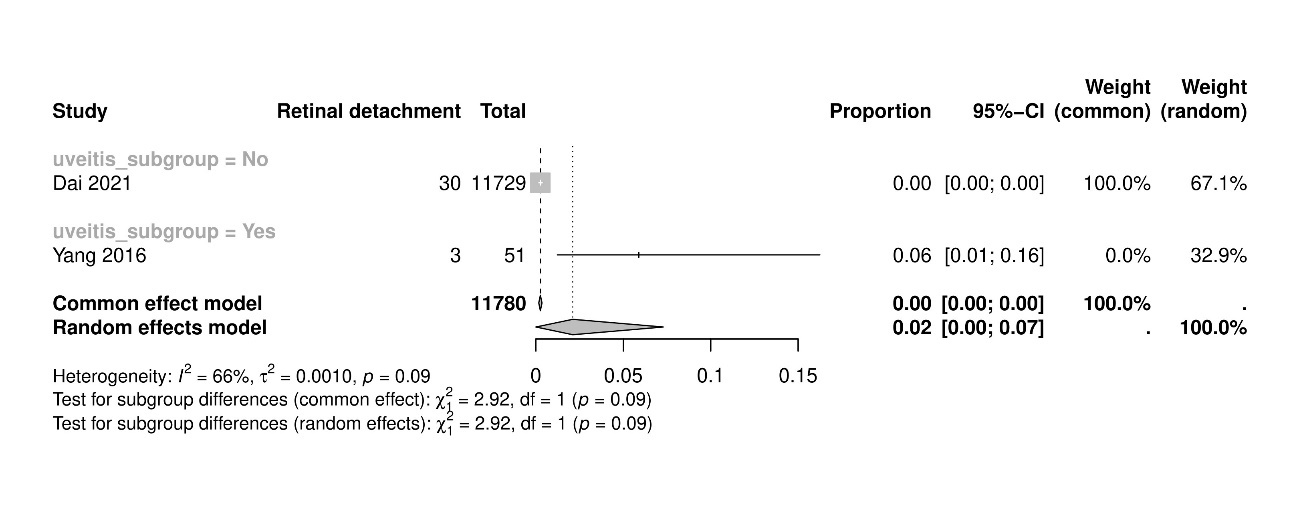


**Supplementary Material Figure 19**
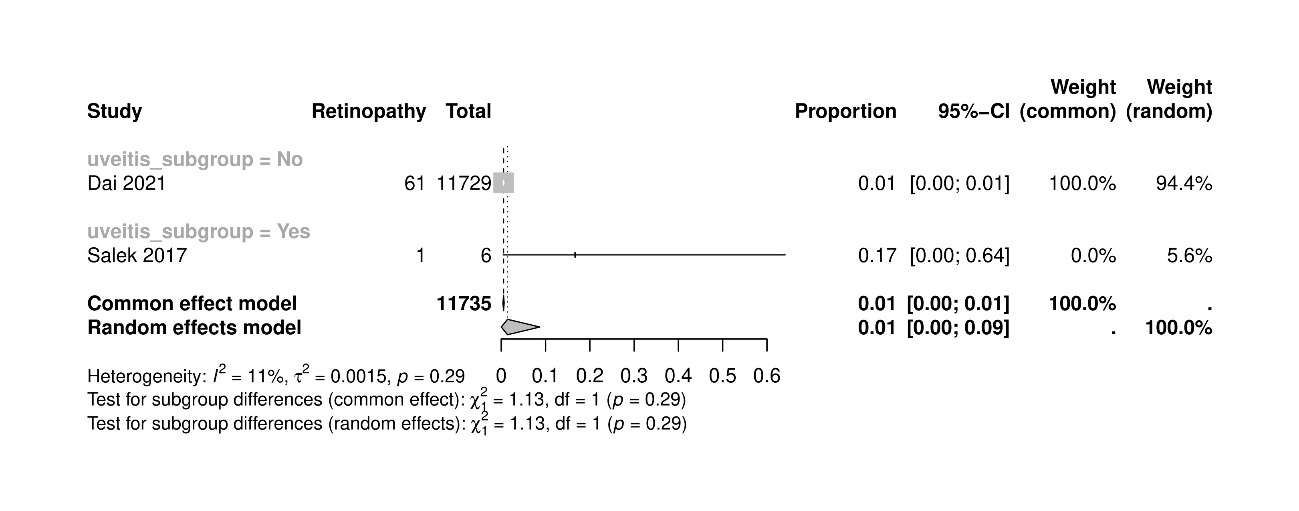


**Supplementary Material Figure 20**
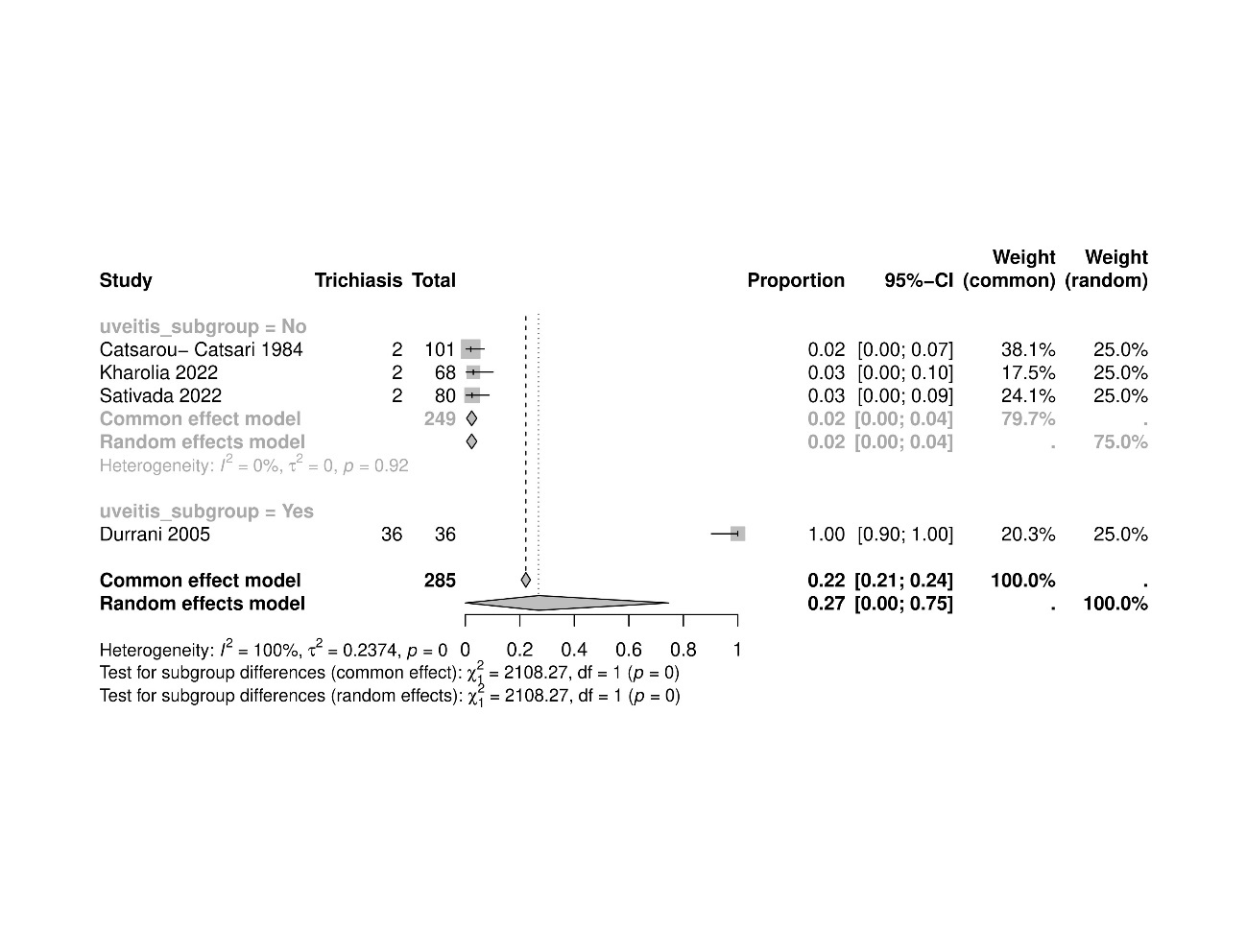


**Supplementary Material Figure 21**
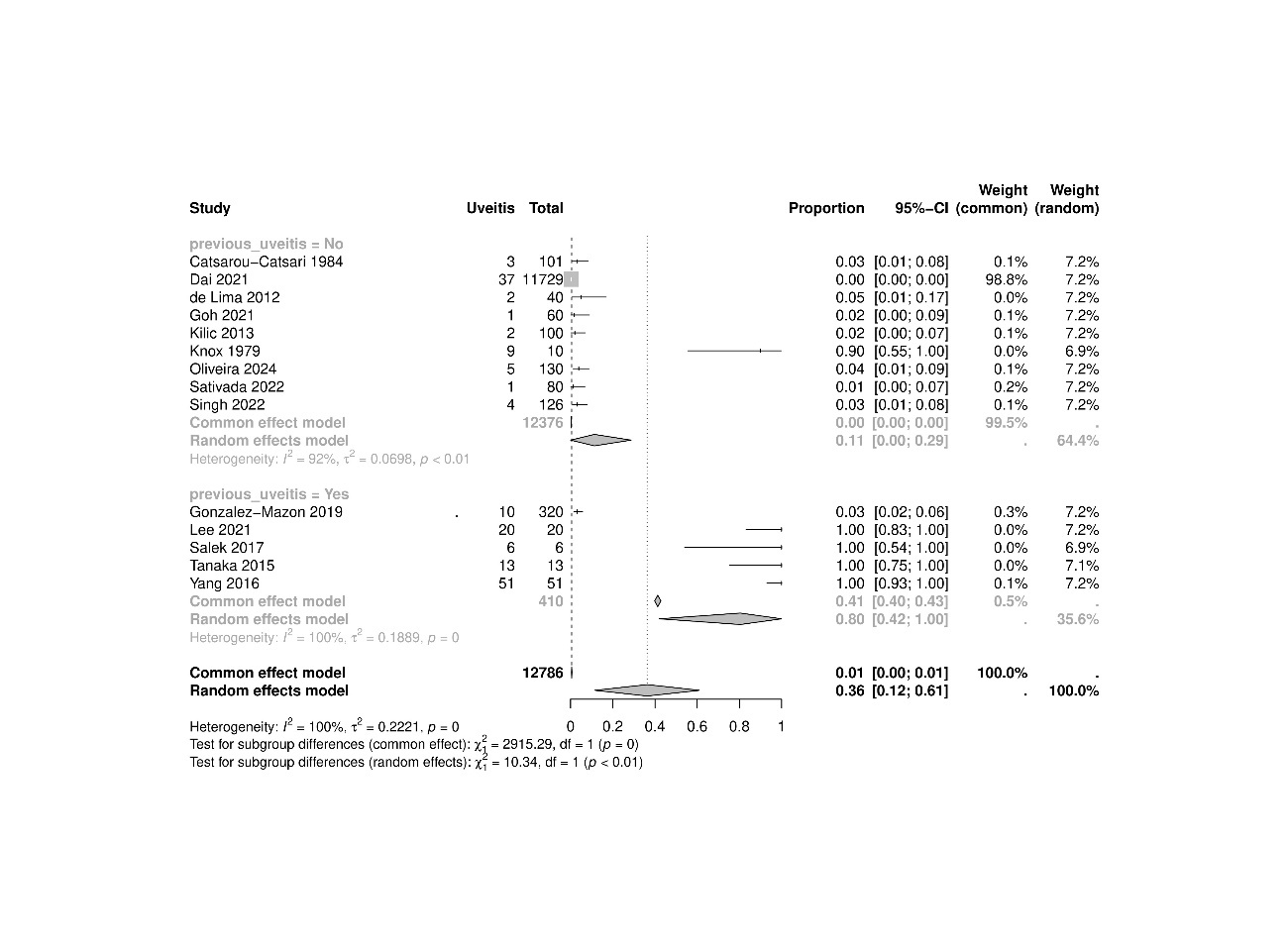


**Supplementary Material Figure 22**
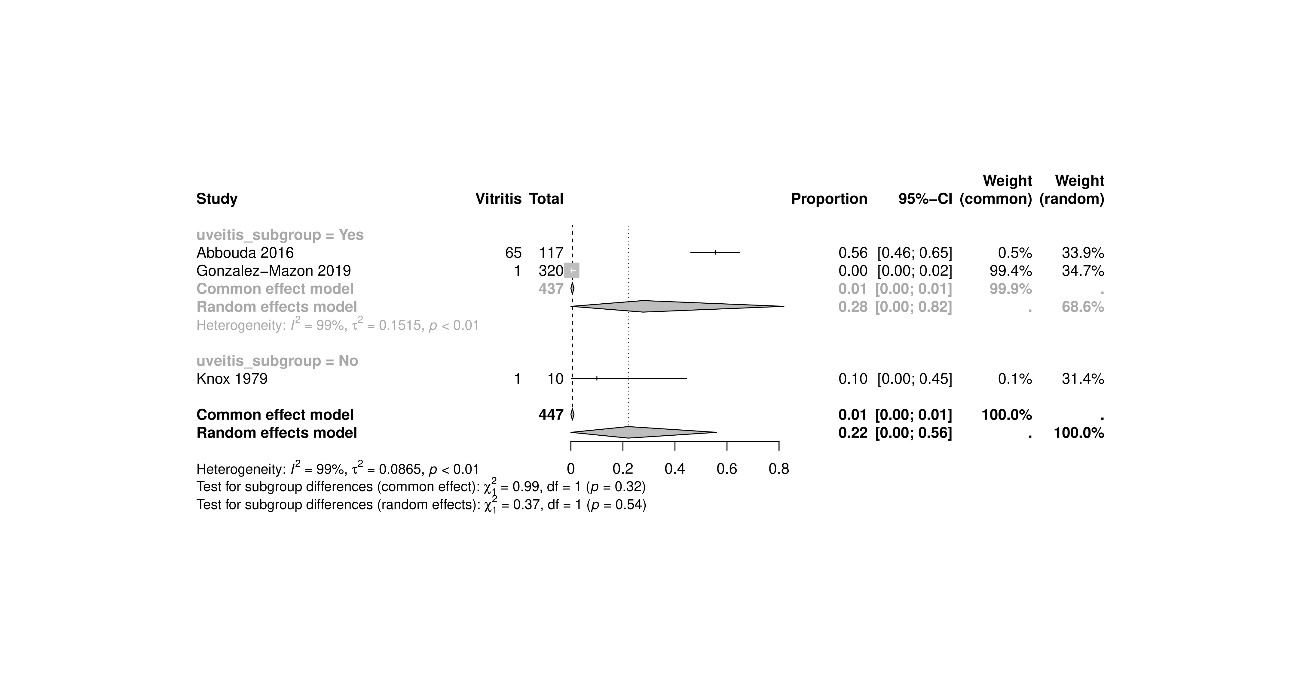


**Supplementary Material Figure 23**
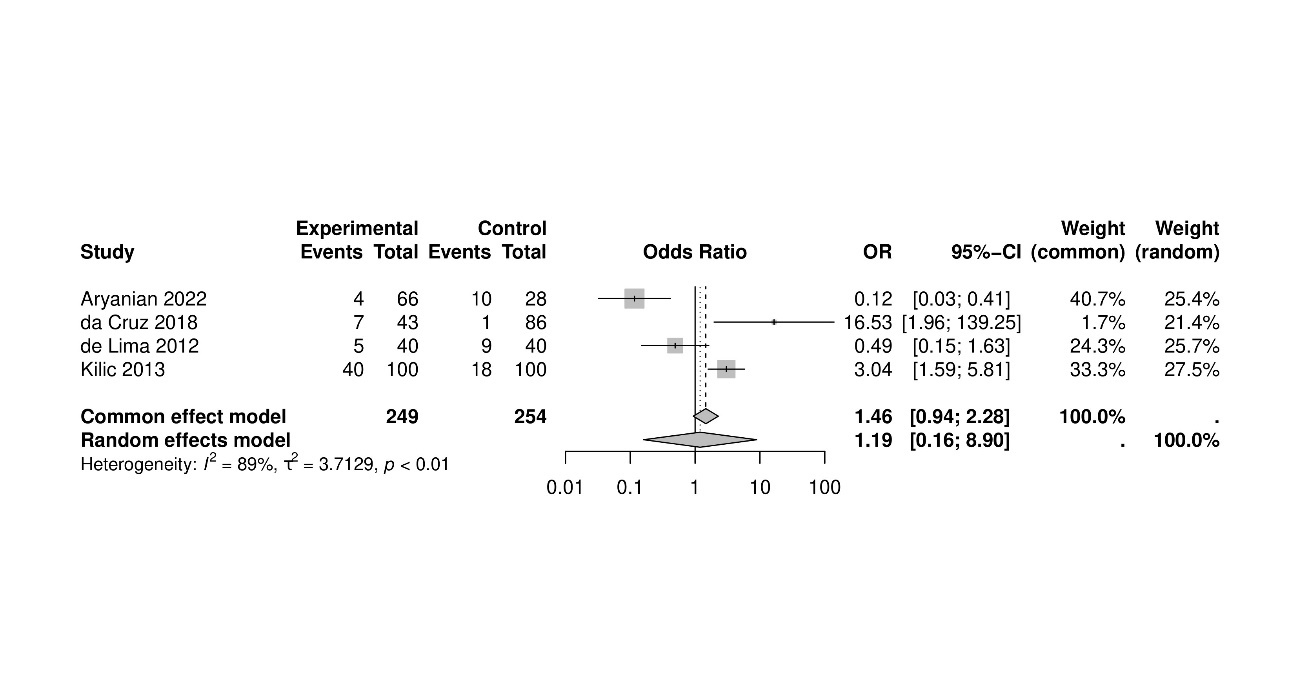


**Supplementary Material Figure 24**
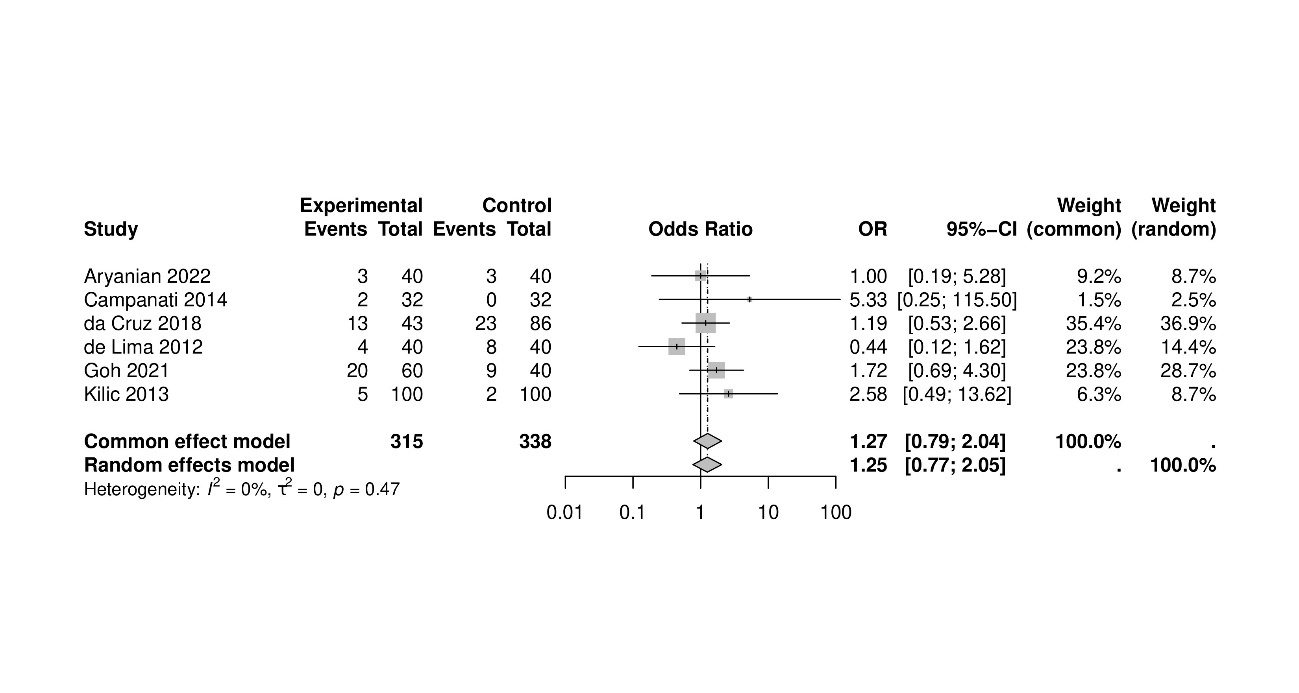


**Supplementary Material Figure 25**
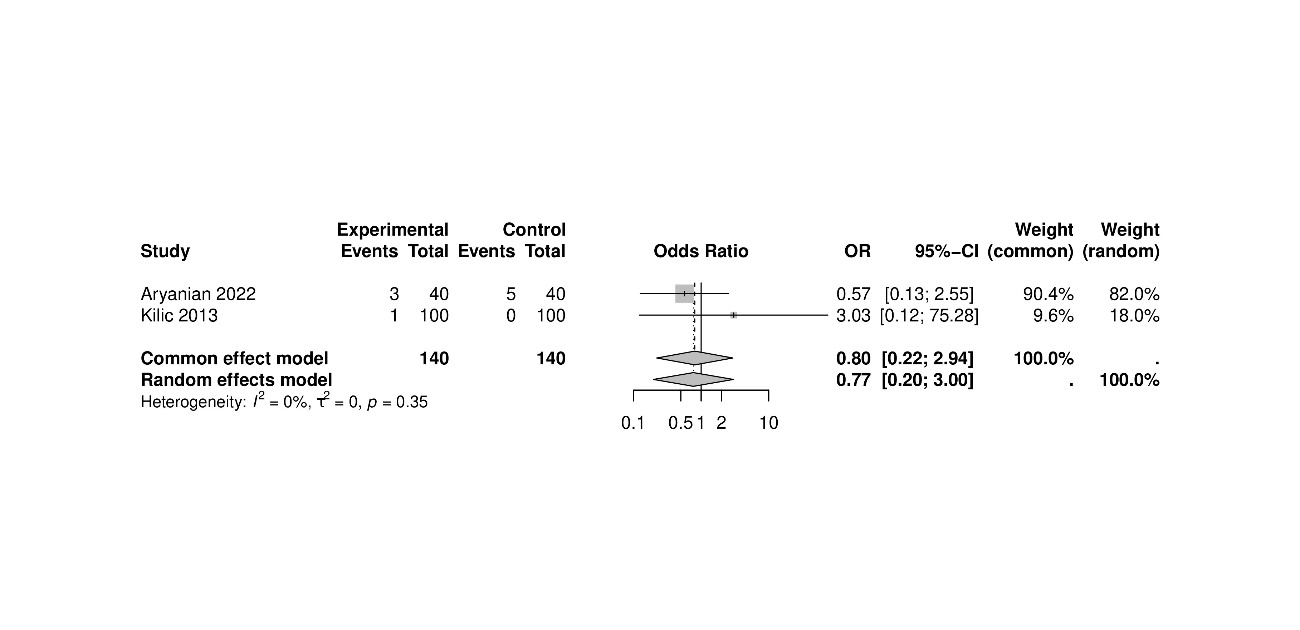


**Supplementary Material Figure 26**
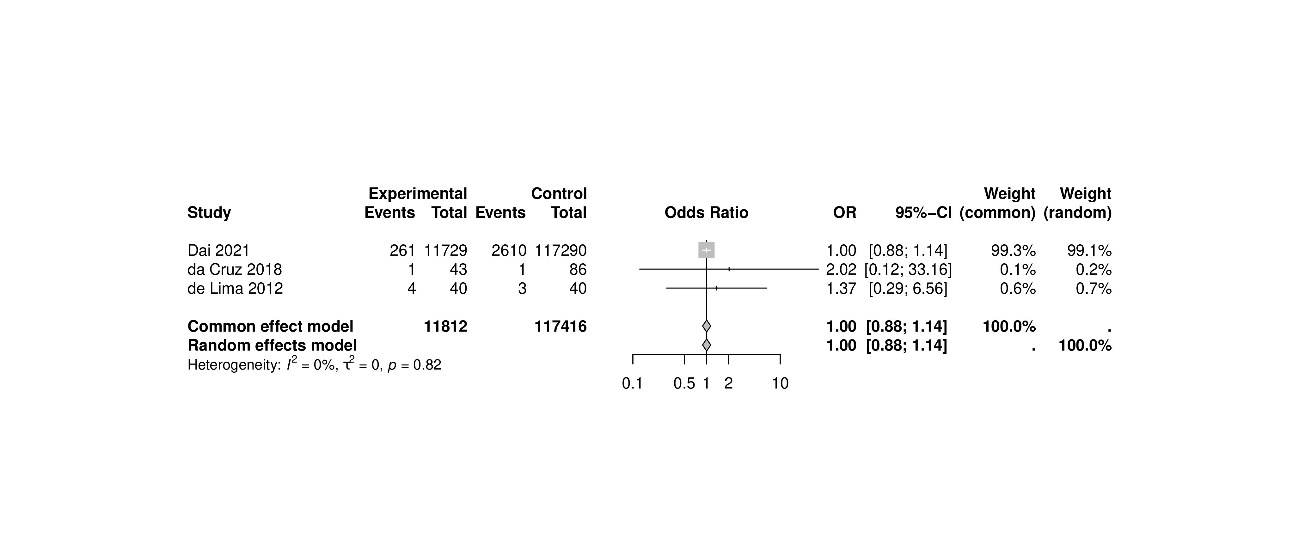


**Supplementary Material Figure 27**
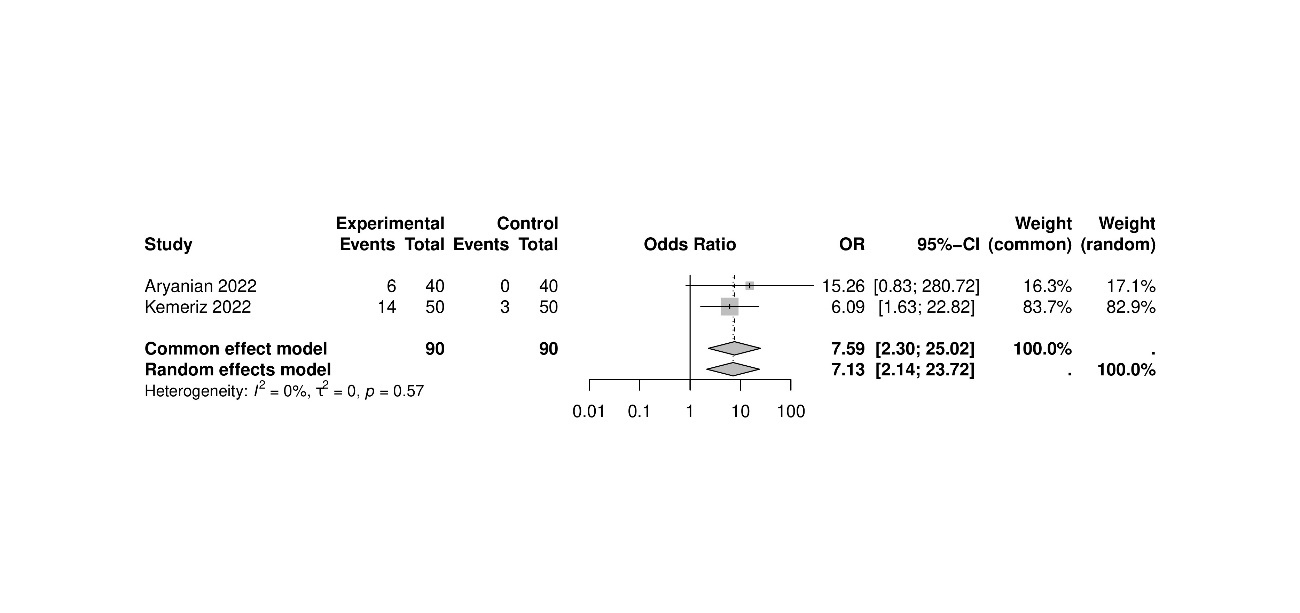


**Supplementary Material Figure 28**
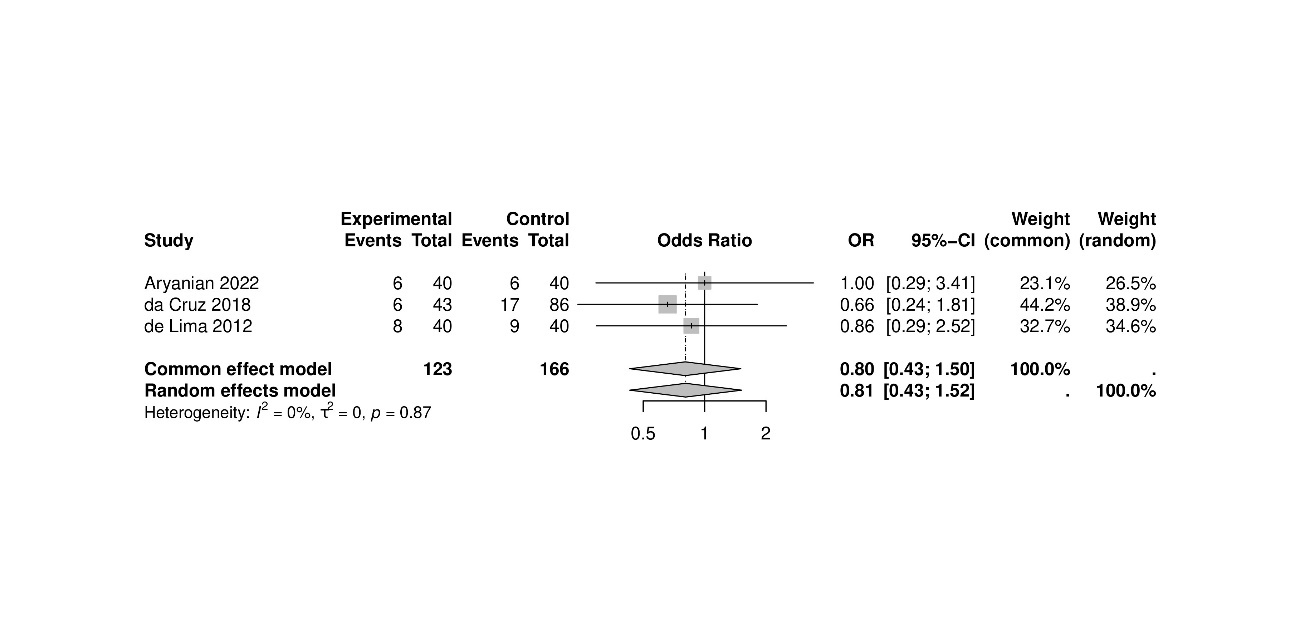


**Supplementary Material Figure 29**
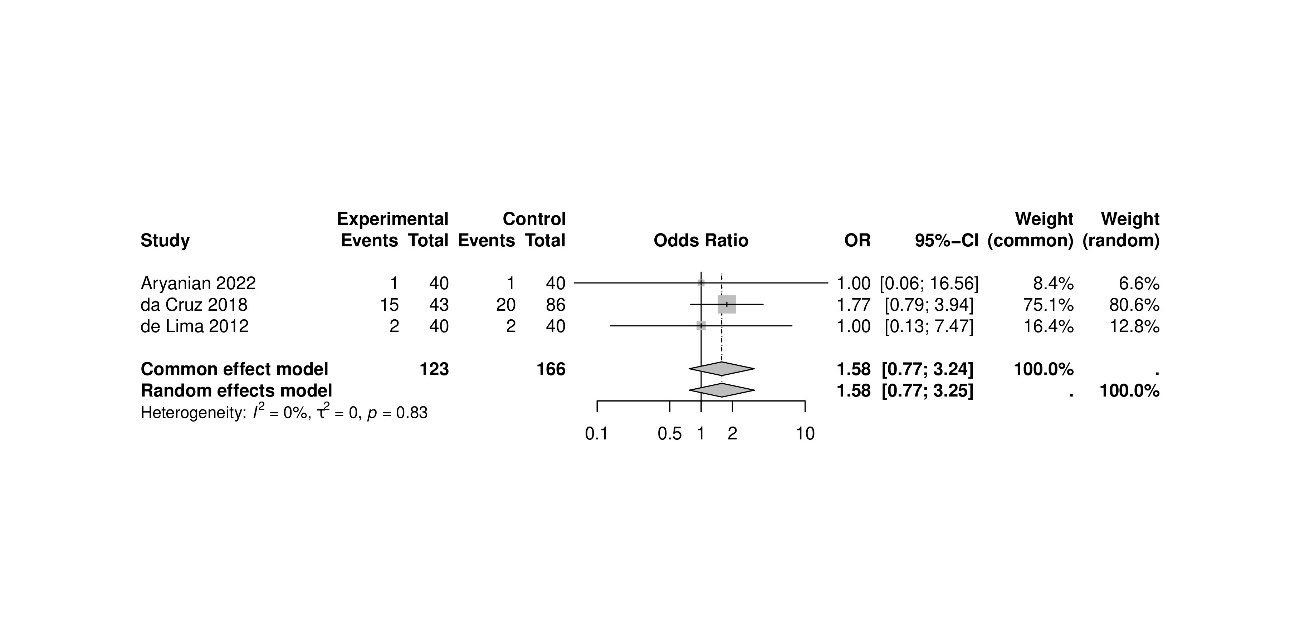


**Supplementary Material Figure 30**
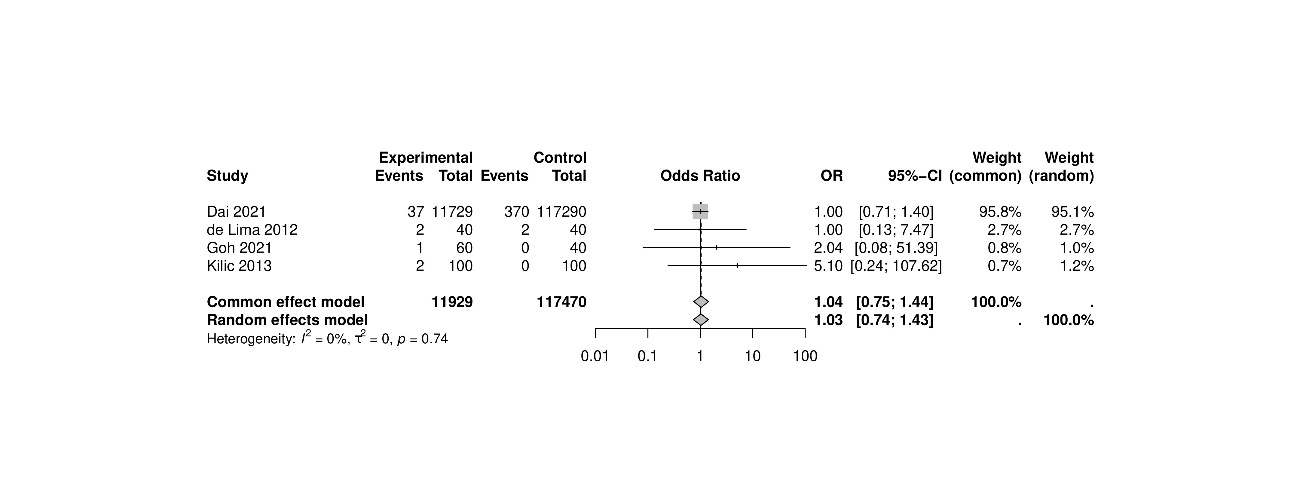

Supplement: Supplementary file 1 — Supplementary Material 1 [file 12348_2025_486_MOESM1_ESM.docx]
